# Blockade of Arf1-mediated lipid metabolism in cancers promotes tumor infiltration of cytotoxic T cells via the LPE-PPARγ-NF-κB-CCL5 pathway

**DOI:** 10.1093/lifemeta/load036

**Published:** 2023-09-06

**Authors:** Na Wang, Tiange Yao, Chenfei Luo, Ling Sun, Yuetong Wang, Steven X Hou

**Affiliations:** Department of Cell and Developmental Biology at School of Life Sciences, State Key Laboratory of Genetic Engineering, Institute of Metabolism and Integrative Biology, Human Phenome Institute, Fudan University, Shanghai 200438, China; Department of Cell and Developmental Biology at School of Life Sciences, State Key Laboratory of Genetic Engineering, Institute of Metabolism and Integrative Biology, Human Phenome Institute, Fudan University, Shanghai 200438, China; Department of Cell and Developmental Biology at School of Life Sciences, State Key Laboratory of Genetic Engineering, Institute of Metabolism and Integrative Biology, Human Phenome Institute, Fudan University, Shanghai 200438, China; Department of Cell and Developmental Biology at School of Life Sciences, State Key Laboratory of Genetic Engineering, Institute of Metabolism and Integrative Biology, Human Phenome Institute, Fudan University, Shanghai 200438, China; Department of Cell and Developmental Biology at School of Life Sciences, State Key Laboratory of Genetic Engineering, Institute of Metabolism and Integrative Biology, Human Phenome Institute, Fudan University, Shanghai 200438, China; Department of Cell and Developmental Biology at School of Life Sciences, State Key Laboratory of Genetic Engineering, Institute of Metabolism and Integrative Biology, Human Phenome Institute, Fudan University, Shanghai 200438, China

**Keywords:** immune checkpoint blockade, cancer stem cells, cytotoxic T cells, PPARγ, CCL5-CCR5 pathway, Arf1

## Abstract

Tumor immunotherapy has achieved breakthroughs in a variety of tumors. However, the systemic absence of T cells in tumors and immunosuppressive tumor microenvironment so far limits the efficacy of immunotherapy to a small population of patients. Therefore, novel agents to increase T-cell tumor infiltration are urgently needed in the clinic. We recently found that inhibition of the ADP-ribosylation factor 1 (Arf1)-mediated lipid metabolism not only kills cancer stem cells (CSCs) but also elicits an anti-tumor immune response. In this study, we revealed a mechanism that targeting Arf1 promotes the infiltration of cytotoxic T lymphocytes (CTLs) into tumors through the C-C chemokine ligand 5 (CCL5)- C-C chemokine receptor type 5 (CCR5) pathway. We found that blockage of Arf1 induces the production of the unsaturated fatty acid (PE 18:1) that binds and sequestrates peroxisome proliferator-­activated receptor-γ (PPARγ) from the PPARγ-nuclear factor-κB (NF-κB) cytoplasmic complex. The released NF-κB was then phosphorylated and translocated into the nucleus to regulate the transcription of chemokine CCL5. CCL5 promoted infiltration of CTLs for tumor regression. Furthermore, the combination of the Arf1 inhibitor and programmed cell death protein 1 (PD-1) blockade induced an even stronger anti-tumor immunity. Therefore, targeting Arf1 represents a novel anti-tumor immune approach by provoking T-cell tumor infiltration and may provide a new strategy for tumor immunotherapy.

## Introduction

Recent revolutionized success of cancer immunotherapy using checkpoint blockade (ICB) suggests that it is likely to form the foundation of future curative therapy for many cancers [1, 2]. However, the response rate of patients to ICB remains relatively low in most cases [3]. The paucity of immune cells in tumors remains a major cause and is correlated with poor cancer survival [4]. In melanoma, patients infiltrated by a large number of tumor antigen-specific CD8^+^ T cells, referred to as a “hot tumor”, respond well to ICBs. In contrast, patients with insufficient specific CD8^+^ T-cell infiltration of their tumor, a characteristic known as “cold tumor”, have limited responses to ICBs [5]. Therefore, increasing tumor antigen-specific CD8^+^ T-cell infiltration would be a good strategy to improve immunotherapy. The tumor microenvironment (TME) significantly affects the infiltration of T cells into the tumors [6, 7]. There is a major ongoing effort to improve immunotherapy through alternating TME [6]. Cancer cells are major players in modulating the TME [8]. Tumor cells were found to dictate immune responses in their microenvironment by producing chemokines [9]. Tumor cells often escape from the immune system by creating an immunosuppressive milieu [6]. Stem cell-like subpopulations existing in solid tumors have been found to maintain tumor niche capable of immunosuppression and therapeutic evasion [10]. In triple-negative breast cancer, it was recently found that quiescent cancer stem cells (CSCs) constitute an immunosuppressive niche by orchestrating a local hypoxic immune-suppressive milieu that is characterized by dysfunctional dendritic cells (DCs), suppressive fibroblasts, reduced T-cell infiltration, and enhanced T-cell exhaustion [11]. The development of strategies to eradicate cancer stem cells (CSCs) and to change their immune-suppressive environment will be key to improving responses to immunotherapy in cancer treatments.

ADP-ribosylation factor 1 (Arf1) is a member of the human Arf gene family, which encodes small guanine nucleotide-binding proteins and plays an important role in vesicular trafficking [12–14]. Previous investigations have demonstrated that Arf1 is highly expressed in multiple human cancers, including breast cancer, hepatocellular cancer, colorectal cancer, and prostate cancer [15, 16]. Arf1 upregulation is negatively correlated with the prognosis of patients with cancer [15, 17].

We recently found that ablation of the Arf1-mediated lipid metabolism not only kills CSCs but also elicits an anti-tumor immune response [15]. In this study, we found that knockdown of Arf1 in tumor cells or treatment of mice with the Arf1 inhibitors dramatically suppresses tumor formation by promoting tumor infiltration of cytotoxic T cells via the lyso-phosphatidyl ethanolamine (LPE)-peroxisome proliferator-activated receptor-γ (PPARγ)-nuclear factor-κB (NF-κB)-C-C chemokine ligand 5 (CCL5) pathway. Further, we demonstrated that the Arf1 inhibitors and programmed cell death protein 1 (PD-1) blockade reduce tumor growth synergistically. Therefore, the new Arf1 inhibitors represent a new type of anti-tumor immune reagents and can complement and improve current tumor immunotherapy.

## Results

### Arf1 ablation in tumor cells promotes cytotoxic T-cell infiltration

**Figure 1:**
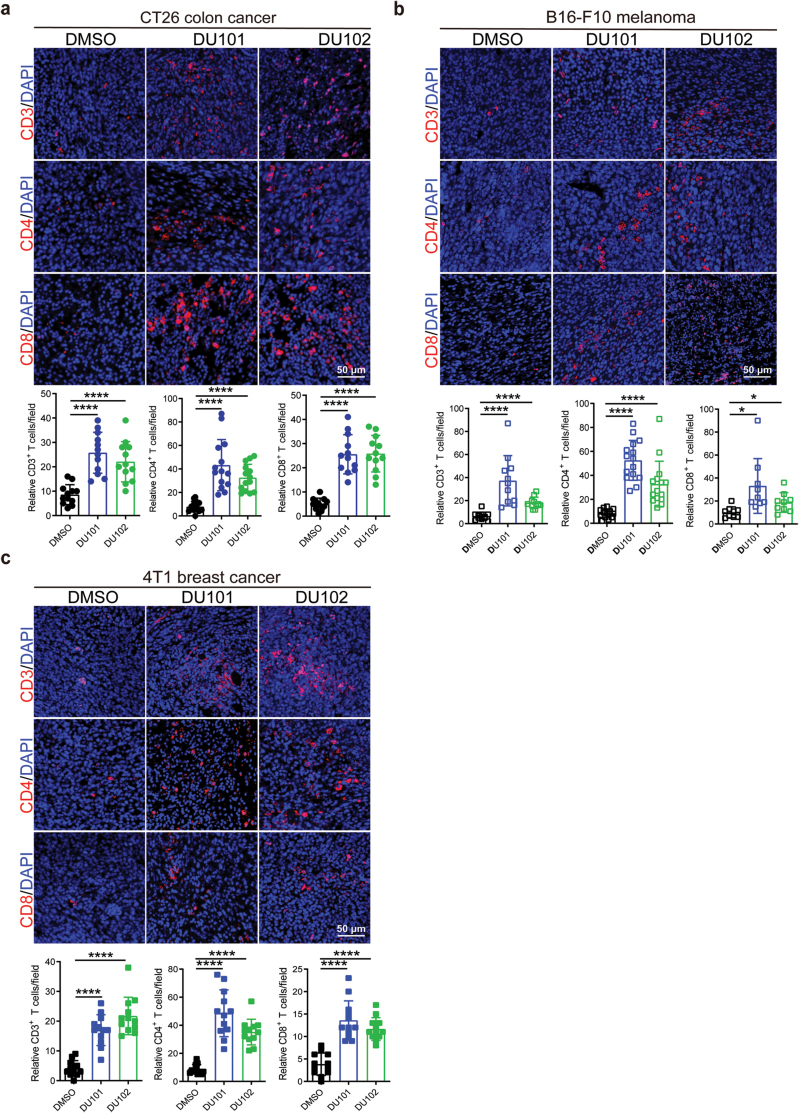
The Arf1 inhibitors enhance infiltration of T cells into tumors. (a) IF staining and quantification of CD3^+^, CD4^+^, and CD8^+^ T cells in CT26 colon tumors that were treated with DMSO, DU101, or DU102 (*n* = 15 in each group). (b) IF staining and quantification of CD3^+^, CD4^+^, and CD8^+^ T cells in B16-F10 melanoma that were treated with DMSO, DU101, or DU102 (*n* = 15 in each group). (c) IF staining and quantification of CD3^+^, CD4^+^, and CD8^+^ T cells in 4T1 breast tumors that were treated with DMSO, DU101, or DU102 (*n* = 15 in each group). Data are shown as mean ± SEM. Student’s *t* test. ^*^P < 0.05, ^**^P < 0.01, ^****^P < 0.0001.

We previously reported that Arf1 ablation in CSCs promoted anti-tumor immunity in mice [15]. To study the effect of Arf1 ablation on T-cell infiltration into tumors, we performed immuno­fluorescence (IF) staining and flow cytometric analysis in tumors treated with two newly developed Arf1 inhibitors (DU101 and DU102, details on the new inhibitors were described in Patent No. PCT/CN2021/110373 and a separate manuscript that is in submission) and found increased T-cell infiltration in CT26 allograft (Fig. 1a, Supplementary Fig. S1a–c), B16-F10 allograft (Fig. 1b, Supplementary Fig. S1g–i), and 4T1 allograft (Fig. 1c, Supplementary Fig. S1d–f). Consistently, T-cell infiltration was also dramatically enhanced in allograft tumors with Arf1 knockdown (Supplementary Fig. S1j–l).

### Targeting Arf1 recruits cytotoxic T cells by inducing CCL5 transcription

**Figure 2:**
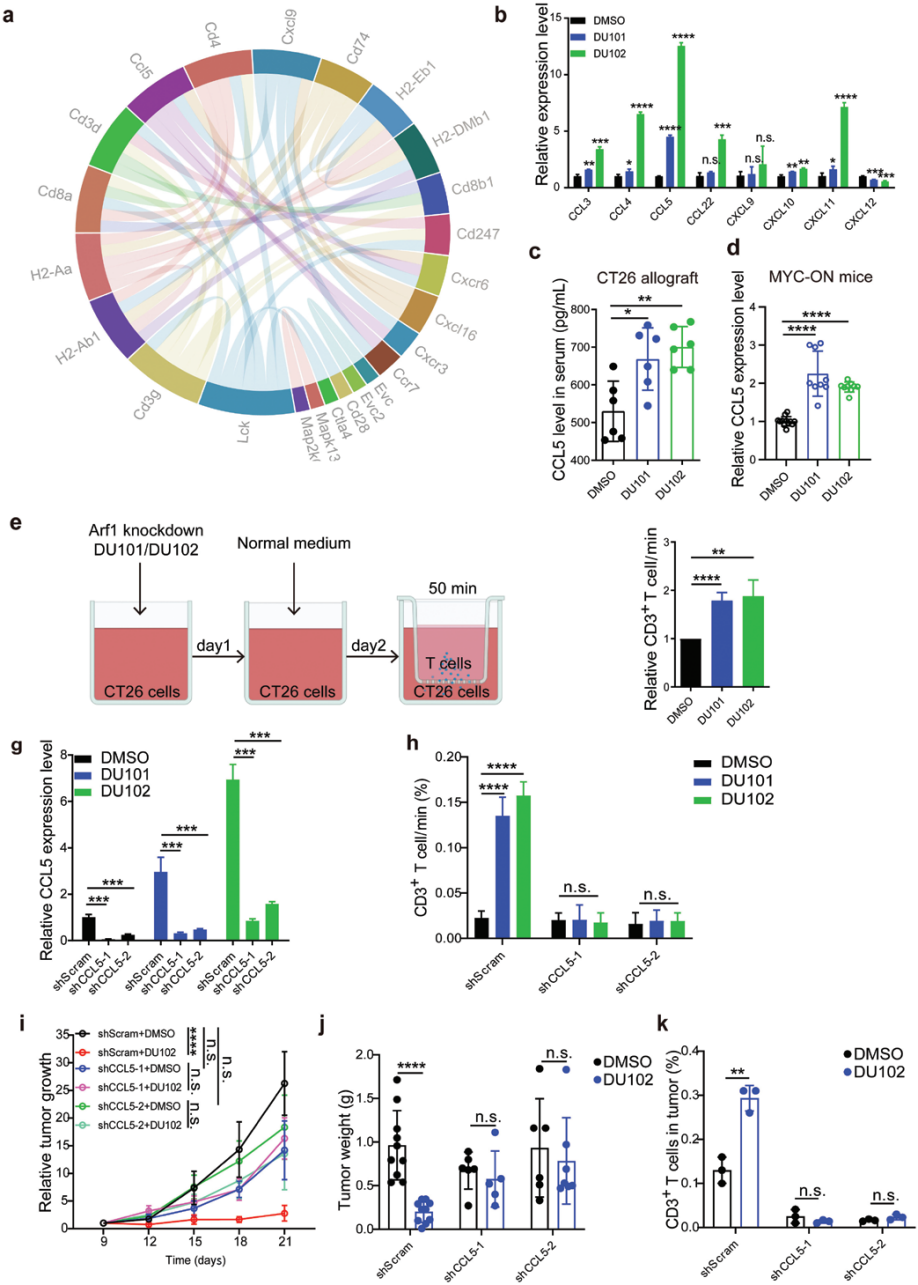
The Arf1 inhibitors promote T-cell infiltration by upregulating CCL5 chemokine. (a) Chord diagram of gene correlation in the Arf1-ablated MYC-ON liver tumors (*n* = 4 in each group). (b) qRT-PCR for chemokines in CT26 cells treated with DMSO, DU101, or DU102. (c) Serum CCL5 levels in the CT26 allografts with the indicated treatments (*n* = 6 in each group). (d) CCL5 mRNA levels in the liver tumors of MYC-ON mice that were treated with DMSO, DU101, or DU102 (*n* = 9 in each group). (e) Experimental design for the co-culture system. (f) FACS analysis of the migration of CD3^+^ T cells in CT26 cells that were treated with DMSO, DU101, or DU102. (g) The mRNA levels of CCL5 in CT26 cells with the indicated knockdowns and treatments. (h) FACS analysis of CD3^+^ T-cell migration in CT26 cells with the indicated knockdowns and treatments. (i and j) Tumor volumes (i) and tumor weights (j) of CT26 allografts with the indicated knockdowns and treatments (*n* = 10 in each group). (k) Percentages of infiltrated CD3^+^ T cells in CT26 allografts with the indicated knockdowns and treatments (*n* = 3 in each group). Data are shown as mean ± SEM. Student’s *t* test. ^*^P < 0.05, ^**^P < 0.01, ^***^P < 0.001, ^****^P < 0.0001. n.s., no significance.

Chemokines play essential roles in orchestrating the migration and differentiation of immune cells [18, 19]. To investigate the mechanism that regulates T-cell infiltration into the Arf1-deficient tumors, we first performed bulk RNA sequence with tumor samples from MYC-ON live tumor mouse models with or without Arf1-knockout. Our results revealed that Arf1 depletion affected multiple immune-related genes associated with chemokines and their receptors (Fig. 2a). CCL5 was also dramatically upregulated in CT26 cells treated with either Arf1 inhibitors or with Arf1 knockdown (Fig. 2b, Supplementary Fig. S2a). Chemokines attract lymphocytes through secretion. With treatment of the Arf1 inhibitors, we observed increased CCL5 levels in the serum of mice bearing CT26 tumor (Fig. 2c) and upregulated mRNA level of CCL5 in MYC-ON mouse liver cancer (Fig. 2d). However, DU101 or DU102 treatment had no impact on the CCL5 level of serum in the Arf1-deficient CT26 allografts (Supplementary Fig. S2b). These results together suggest that Arf1 inhibition in tumor cells induces CCL5 transcription and secretion.

We also performed migration assay of CD3^+^ T cells toward tumor cells (Fig. 2e). We found that the conditional medium from the Arf1-inhibitor-treated CT26 cells had higher capability to promote T-cell migration than that from the DMSO-treated CT26 cells (Fig. 2f). However, the increased capability of promoting T-cell migration was abolished after CCL5 knockdown in the Arf1-inhibitor-treated CT26 cells (Fig. 2g and h). These results were further confirmed in Arf1-deficient CT26 cells (Supplementary Fig. S2c–e).

We further evaluated the role of CCL5 in the Arf1 ­inhibitor-induced antitumor effect. We treated either shScram-treated or shCCL5-treated CT26 allografts with the Arf1 inhibitors and found that DU102 could robustly suppress tumor growth of the shScram-treated CT26 allografts but not the shCCL5-treated CT26 allografts (Fig. 2i and j, Supplementary Fig. S2f). Additionally, CCL5 knockdown in tumor cells also prevented DU102-induced tumoral infiltration of T cells *in vivo* (Fig. 2k). These results were further confirmed in Arf1-deficient CT26 cells (Supplementary Fig. S2g–j). Thus, these data demonstrated that Arf1 inhibition promotes T-cell tumoral infiltration via chemokine CCL5.

### Arf1 inhibition promotes T-cell migration through the CCL5-CCR5 (C-C chemokine receptor type 5) axis

**Figure 3:**
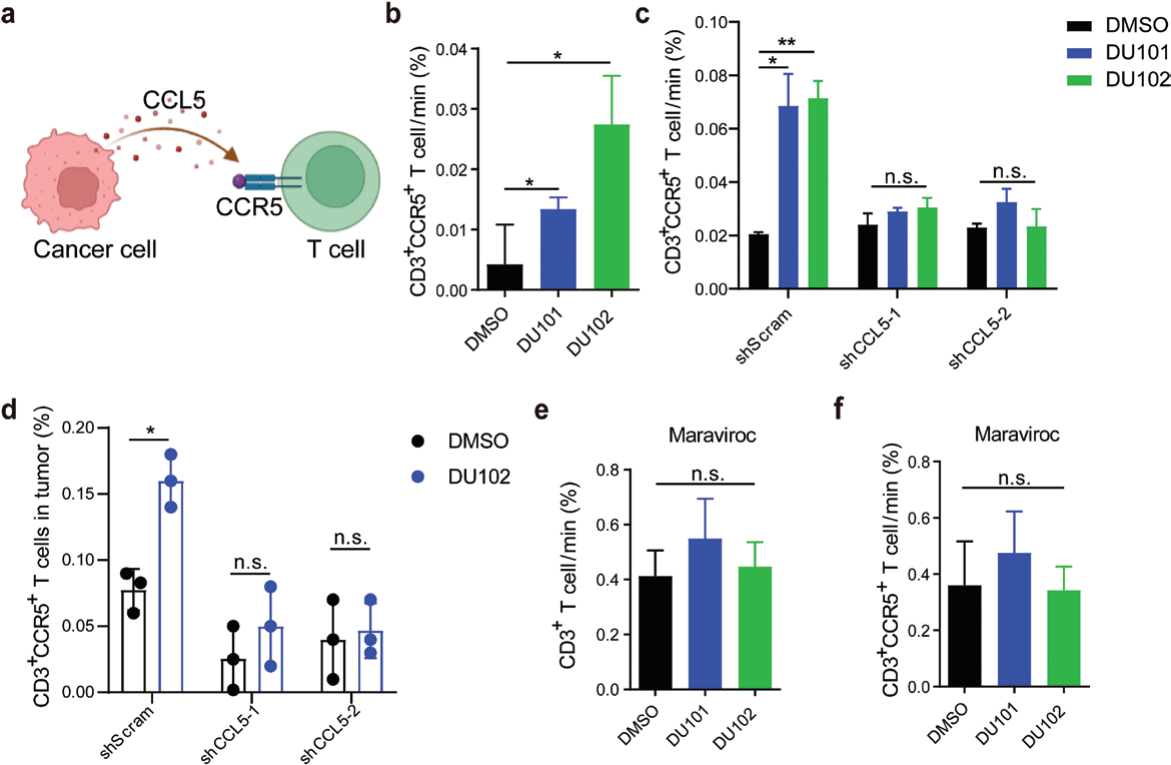
The Arf1 inhibitors promote T-cell infiltration through the CCL5-CCR5 pathway. (a) Schematic interaction between tumor-produced CCL5 and CCR5 on T cells. (b) FACS analysis of proportions of the infiltrated CD3^+^CCR5^+^ T cells in CT26 cells treated with DMSO, DU101, and DU102. (c) FCAS analysis of the infiltrated CD3^+^CCR5^+^ T cells in CCL5-deficient CT26 cells with the indicated treatments. (d) The percentage of infiltrated CD3^+^CCR5^+^ T cells in CT26 allografts with the indicated knockdowns and treatments (*n* = 3 in each group). (e and f) The percentages of the infiltrated CD3^+^ T cells (e) and CD3^+^CCR5^+^ T cells (f) in CT26 cells treated with Arf1 inhibitors and Maraviroc, a CCR5 antagonist. Data are shown as mean ± SEM. Student’s *t* test. ^*^P < 0.05, ^**^P < 0.01. n.s., no significance.

Chemokine CCL5 interacts with several receptors on T cells, including CCR1, CCR3, CCR4, and CCR5, while CCR5 showed the highest affinity with CCL5 [20–22] (Fig. 3a). In the *in vitro* migration assay, we found that CD3^+^CCR5^+^ T-cell migration was significantly enhanced toward CT26 cells treated with the two Arf1 inhibitors, DU101 and DU102 (Fig. 3b, Supplementary Fig. S3a). However, Arf1 inhibitor-induced migration of CD3^+^CCR5^+^ T cells was significantly abolished after CCL5 knockdown *in vitro* and *in vivo* (Fig. 3c and d, Supplementary Fig. S3b and c). To further investigate the function of the CCL5-CCR5 axis in recruiting CD3^+^ T cells, we pre-treated T cells with Maraviroc, a small molecule inhibitor of CCR5, and found that the migration of CD3^+^ T cells as well as CD3^+^CCR5^+^ T cells toward the Arf1-ablated CT26 cells was dramatically blocked (Fig. 3e and f, Supplementary Fig. S3d and e). Furthermore, analysis of the Cancer Genome Atlas (TCGA) clinical data demonstrated that the expression level of CCL5 in tumors is positively correlated with CD8^+^ T-cell infiltration and hepatocarcinoma patient survival (Supplementary Fig. S3f and g). These data together demonstrated that Arf1 inhibition suppresses tumor growth by promoting CD3^+^ T-cell infiltration into the tumor though the CCL5-CCR5 axis.

### Arf1 inhibition promotes CCL5 transcription through the LPE-PPARγ axis

**Figure 4:**
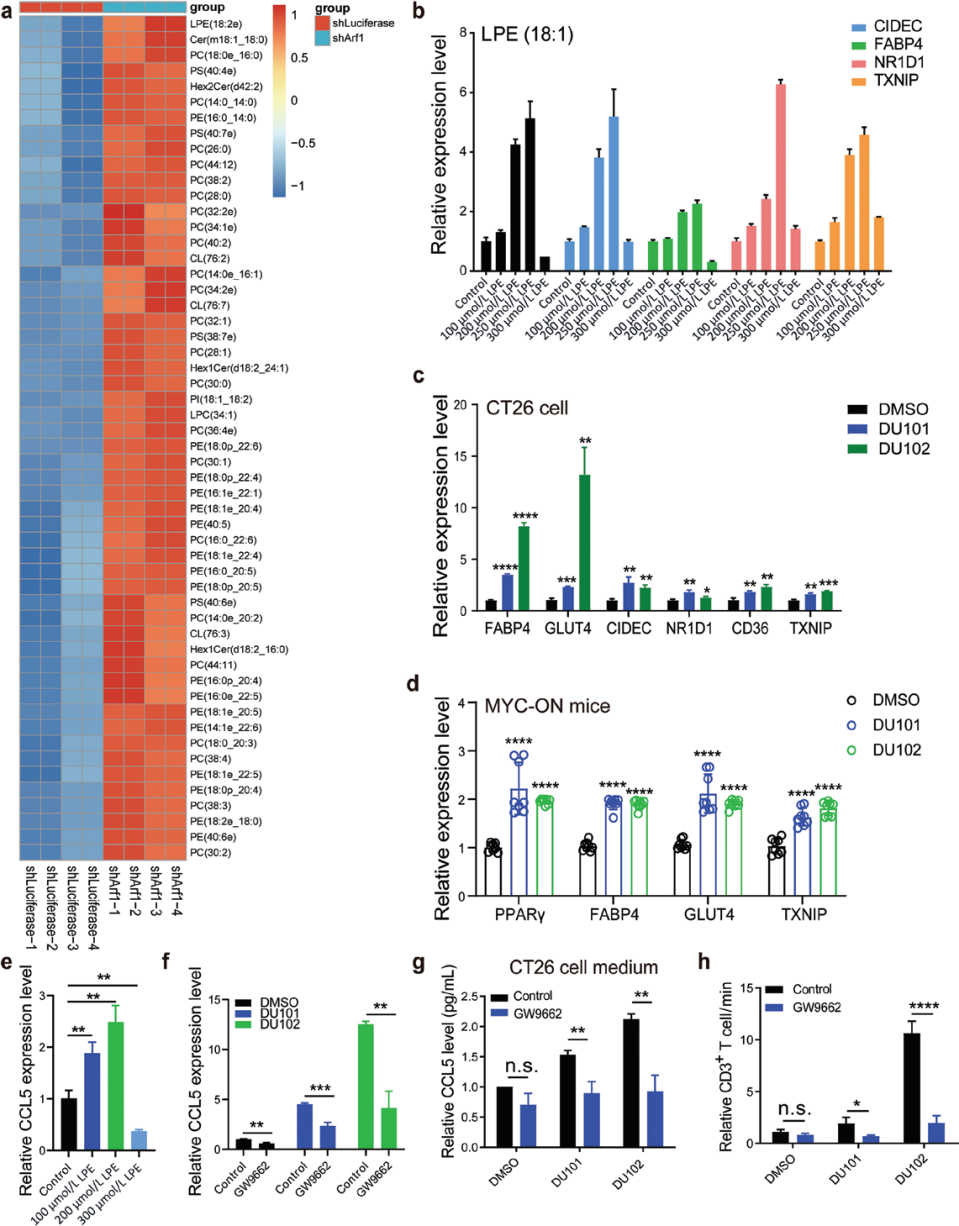
The Arf1 inhibitors activate the PPARγ pathway by inducing unsaturated fatty acid (LPE). (a) Heatmap of different lipid fractions in the Arf1-deficient CT26 cells. (b) The mRNA levels of downstream genes of PPARγ were detected in CT26 cells treated with different concentrations of LPE (0, 100, 200, 250, and 300 μmol/L for 24 h) by qRT-PCR. (c and d) The mRNA levels of PPARγ in CT26 cells (c) and MYC-ON liver tumors (d) treated with DMSO or Arf1 inhibitors were detected by qRT-PCR. (e) The CCL5 mRNA levels in CT26 cells treated with different concentrations of LPE were detected by qRT-PCR. (f) The CCL5 mRNA levels in CT26 cells with vesicle or GW9662 treatment were detected by qRT-PCR. (g) The CCL5 levels in cell medium collected from CT26 cells with the indicated treatments were determined by ELISA. (h) The percentages of the infiltrated CD3^+^ T cells in CT26 cells with the indicated treatments. Data are shown as mean ± SEM. Student’s *t* test. ^*^P < 0.05, ^**^P < 0.01, ^***^P < 0.001, ^****^P < 0.0001. n.s., no significance.

To understand the regulatory mechanism of CCL5 transcription by the Arf1 inhibition, we performed correlation analysis on RNA-sequencing data of patients with liver hepatocellular carcinoma from the TCGA database and found that the PPARγ signaling pathway was the most negatively correlated between normal tissue and tumors (Supplementary Fig. S4a). The activation of the PPARγ signaling in Arf1 inhibitor-treated CT26 cells was confirmed by detecting mRNA expression of its downstream target genes (Fig. 4c, Supplementary Fig. S4c). We also observed enhanced mRNA levels of these downstream target genes of the PPARγ signaling in the MYC-ON mouse liver cancer treated with Arf1 inhibitors (Fig. 4d). PPARγ is a transcription regulator [23–25], which can be activated by unsaturated fatty acids such as phosphatidylcholine (PC) and phosphatidic acid (PA) [26]. We previously reported that the Arf1-mediated lipolysis pathway sustains stem cells and CSCs [15, 27]. We recently found that the Arf1-ablated neurons accumulated lipid droplets and released peroxidized lipids to promote inflammation neurodegeneration in spinal cord and hindbrain [28]. Therefore, lipids generated in the Arf1-ablated cells are likely unsaturated fatty acids.

To identify the connection between Arf1 ablation and PPARγ activation, we performed the lipidomic analysis and found that the single unsaturated fatty acid, phosphatidylethanolamine (PE 18:1), was dramatically elevated and LPE was at the top in the Arf1-deficient CT26 cells in comparison with that in the control CT26 cells (Fig. 4a and Supplementary Fig. S4b). It was previously shown that oxidized PEs (HETE-PEs) activate PPARγ in macrophages and induce CD36 in human monocytes [29, 30]. Based on this information, we selected LPE for further analysis. We then directly treated CT26 cells with the cell-permeable single unsaturated PE (LPE 18:1) to examine whether the unsaturated PE could function as a potential ligand of PPARγ or not. We found that LPE treatment significantly increased expressions of PPARγ target genes in a dose-dependent manner, suggesting that LPE might be a potential ligand of PPARγ (Fig. 4b). Further, we found that LPE treatment also upregulated the mRNA level of CCL5 (Fig. 4e). In addition, we found that inhibiting PPARγ with GW9662, a selective PPARγ antagonist, could significantly downregulate the mRNA level of CCL5 in the Arf1-ablated CT26 cells (Fig. 4f, Supplementary Fig. S4d). GW9662 treatment could also reduce the secreted CCL5 (Fig. 4g, Supplementary Fig. S4e). Furthermore, GW9662 treatment also prevented the migration of CD3^+^ T cells toward the Arf1-ablated tumor cells (Fig. 4h, Supplementary Fig. S4f). These results together indicated that the Arf1 inhibition promotes CCL5 expression through the LPE-PPARγ pathway.

### NF-κB regulates CCL5 transcription in the Arf1-ablated tumor cells

**Figure 5:**
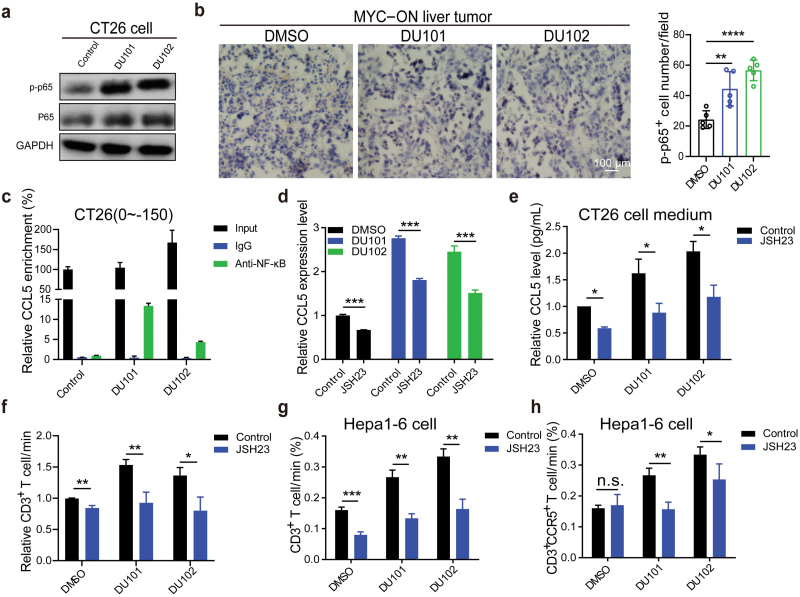
The Arf1 inhibitors promote T-cell tumor infiltration by activating the NF-κB-CCL5 pathway. (a) The phosphorylated p65 in CT26 cells treated with vesicle, DU101, or DU102 was detected by western blot analysis. (b) The phosphorylated p65 in MYC-ON liver tumors after DMSO, DU101, or DU102 treatment was detected by IHC staining. The right was the statistical analysis of p-p65^+^ cells per field. (c) The relative CCL5 enrichment in CT26 cells with the indicated treatments was analyzed by ChIP-PCR assay. (d) The CCL5 mRNA levels in CT26 cells with the indicated treatments were detected by qRT-PCR. (e) The CCL5 levels in the cell medium collected from CT26 cells with the indicated treatments were detected by the mouse CCL5 ELISA kit. (f) FACS analysis of the infiltrated CD3^+^ T cells in CT26 cells with the indicated treatments. (g and h) The proportions of the infiltrated CD3^+^ T cells (g) and CD3^+^CCR5^+^ T (h) cells in Hepa1-6 cells with the indicated treatments were detected by FACS analysis. Data are shown as mean ± SEM. Student’s *t* test. ^*^P < 0.05, ^**^P < 0.01, ^***^P < 0.001, ^****^P < 0.0001. n.s., no significance.

NF-κB was reported to be the main transcription factor of CCL5 [31]. We examined whether NF-κB regulated the transcription of CCL5 or not in tumor cells with Arf1 inhibitor treatment or Arf1 knockdown, and found that inhibiting Arf1 significantly upregulated the phosphorylation level of p65 protein (p-p65) in CT26 cells (Fig. 5a, Supplementary Fig. S5a). In addition, the number of p-p65-positive cells was also obviously increased in MYC-ON mouse liver cancer treated with DU101 or DU102 (Fig. 5b). However, the numbers of p-p65-positive cells have no difference after DU101 or DU102 treatment in the Arf1-deficient CT26 allografts (Supplementary Fig. S5b and c). These data indicated that NF-κB was activated in tumor cells with either Arf1 knockdown or DU101/DU102 treatment. To further assess whether NF-κB directly regulated transcription of CCL5 or not, we performed chromatin immunoprecipitation (ChIP)-PCR and found that either Arf1 knockdown or DU101/DU102 treatment could remarkably increase the binding of NF-κB protein to CCL5 promoter at the position of 150 bp upstream of start codon (Fig. 5c, Supplementary Fig. S5d and e). Treatment with an NF-κB inhibitor, JSH23, could significantly reduce CCL5 expression induced by either Arf1 knockdown or DU101/DU102 treatment (Fig. 5d, Supplementary Fig. S5f). The secreted CCL5 in the culture medium was also reduced after JSH23 treatment (Fig. 5e, Supplementary Fig. S5g). Furthermore, treatment of tumor cells with JSH23 could block tumor migration of the CD3^+^ T cells induced by the Arf1-CCL5 pathway in CT26 cells (Fig. 5f, Supplementary Fig. S5h). Consistently, Arf1 inhibitor-induced migration of CD3^+^ T and CD3^+^CCR5^+^ T cells was also significantly suppressed by JSH23 treatment in Hepa1-6 cells (Fig. 5g and h). Collectively, these data together demonstrated that Arf1 ablation in tumor cells promotes migration of the CD3^+^ T cells through the NF-κB-CCL5-CCR5 pathway.

### Arf1 regulates the interaction of PPARγ and NF-κB through LPE

**Figure 6:**
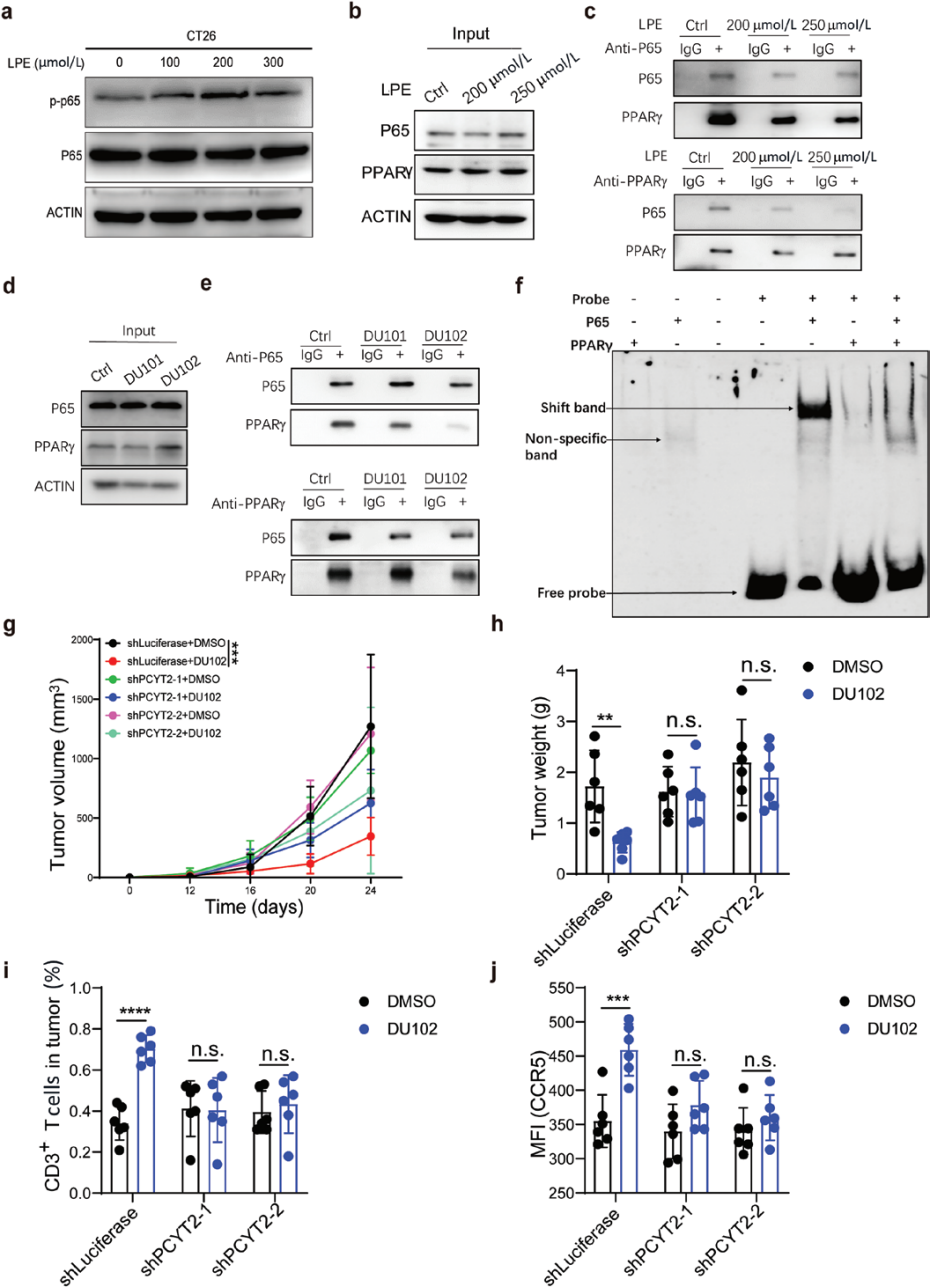
The Arf1 inhibitors disrupt the interaction between PPARγ and NF-κB. (a) The phosphorylated p65 was detected in CT26 cells treated with different concentrations of LPE. (b and c) The interaction between p65 and PPARγ in LPE-treated CT26 cells was detected by Co-IP assay. (d and e) The interaction between PPARγ and p65 in CT26 cells treated with DMSO or Arf1 inhibitors was detected by Co-IP. (f) EMSA to detect binding of the indicated proteins to the CCL5 promoter probe. (g and h) Tumor volumes (g) and tumor weights (h) were measured in CT26 allografts with the indicated knockdowns and treatments (*n* = 6 in each group). (i) The proportions of the infiltrated CD3^+^ T cells were analyzed in CT26 allografts with the indicated knockdowns and treatments by FACS analysis. (*n* = 6 in each group). (j) The mean fluorescence intensity (MFI) of CCR5 in CT26 allografts with the indicated knockdowns and treatments. (*n* = 6 in each group). Data are shown as mean ± SEM. Student’s *t* test. ^**^P < 0.01, ^***^P < 0.001, ^****^P < 0.0001. n.s., no significance.

We then studied the relationship between the activated PPARγ pathway and NF-κB pathway in Arf1-deficient tumor cells. Activation of PPARγ by its potential ligand LPE (18:1) treatment could increase the phosphorylation of p65 protein in a dose-dependent manner (Fig. 6a). Further, we found that PPARγ directly bound p65 and their interaction could be significantly inhibited after LPE treatment in the co-immunoprecipitation (Co-IP) assays (Fig. 6b and c). Arf1 knockdown or DU101/DU102 treatment could also disrupt the interaction between PPARγ and NF-κB (Fig. 6d and e, Supplementary Fig. S6a–c). To directly observe the transcriptional regulation of CCL5 by PPARγ and NF-κB, we performed the electrophoretic mobility shift assay (EMSA). We respectively incubated the biotin-labeled CCL5 oligonucleotide probe with purified PPARγ or NF-κB protein. The shifted band was detected in the lane of the CCL5 probe incubated with p65 protein but not with PPARγ protein, indicating that only p65 protein directly bound to the CCL5 promoter (Fig. 6f). However, we could not detect the shifted band after incubation of the biotin-labeled CCL5 probe with purified PPARγ and p65 proteins together, suggesting that PPARγ protein and CCL5 probe may compete binding with p65 protein (Fig. 6f). In conclusion, these results together revealed that Arf1 inhibition first induced unsaturated fatty acid (PE 18:1) that sequested PPARγ from the PPARγ-p65 complex, and then the released p65 moved to nuclei for regulating CCL5 transcription.

To further assess whether PE participated in Arf1 ablation-­induced tumor suppression or not, we generated tumor cells with CTP:phosphoethanolamine cytidylyltransferase (PCYT2) knockdown, which catalyzes the synthesis of PE from ethanolamine. We found that tumor suppression induced by the Arf1 inhibitor treatment was significantly abolished in the PCYT2-deficient CT26 allografts (Fig. 6g and h, Supplementary Fig. S6d), while the body weights of mice showed no difference (Supplementary Fig. S6e). We also found that tumor infiltration of CD3^+^ T and CD8^+^ T cells, CCR5 expression, and T-cell activation in tumors were all significantly reduced after PCYT2 knockdown (Fig. 6i and j, Supplementary Fig. S6f and g). Thus, these data together suggested that the LPE-PPARγ-NF-κB-CCL5 pathway mediates the tumoral infiltration of T cells and tumor suppression induced by the Arf1 inhibitors.

### The Arf1 inhibitor and PD-1 blockade have a synergistic anti-tumor effect

**Figure 7:**
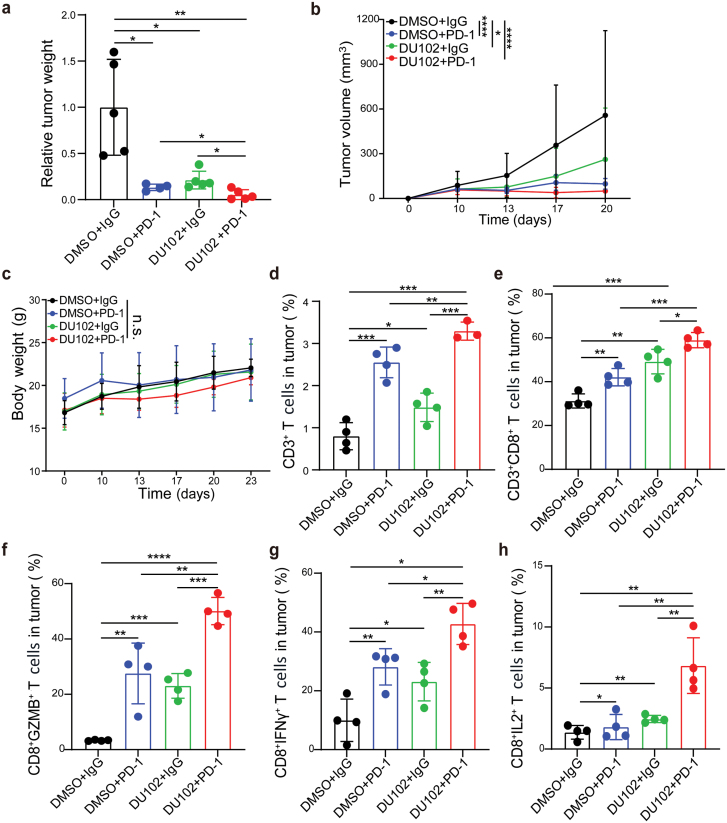
DU102 enhances the anti-tumor effect of anti-PD-1 antibody. (a) Relative tumor weight of mice subcutaneously inoculated with B16-F10 cells and the indicated treatments (*n* = 5 in each group). (b) Tumor volume of B16-F10 allograft treated with vehicle or the indicated reagents. (c) Body weights of mice subcutaneously inoculated with B16-F10 cells and the indicated treatments (*n* = 5 in each group). (d–h) FACS analysis of the percentages of CD3^+^ T cells (d), CD3^+^CD8^+^ T cells (e), CD8^+^GZMB^+^ T cells (f), CD8^+^IFNγ^+^ T cells (g), and CD8^+^IL-2^+^ T cells (h) in tumors with the indicated treatments. Data are shown as mean ± SEM. Student’s *t* test. ^*^P < 0.05, ^**^P < 0.01, ^***^P < 0.001. n.s., no significance.

Since the Arf1 inhibitor treatment promoted T-cell tumor infiltration and the ICBs modulate T-cell activation, their combination may have a synergistic effect. Since melanoma had been proven to respond to the anti-PD-1 treatment well, we investigated their combining effect in the B16-F10 allograft model (Supplementary Fig. S7a). The combination of DU102 (5 mg/kg) and anti-PD-1 antibody showed significantly enhanced anti-­tumor activity compared to that of either DU102 or anti-PD-1 antibody alone (Fig. 7a and b, Supplementary Fig. S7b and c), without affecting body weights and spleen weights of the mice (Fig. 7c, Supplementary Fig. S7d). A combination of DU102 and anti-PD-1 has higher numbers of tumor-infiltrated CD3^+^ T cells, CD3^+^CD8^+^ T cells, and CD3^+^CCR5^+^ T cells in B16-F10 tumors than those in B16-F10 tumors treated by either DU102 or anti-PD-1 alone (Fig. 7d and e, Supplementary Fig. S7f), while there was no difference in tumor-infiltrated CD3^+^CD4^+^ T cells (Supplementary Fig. S7e). The production of Granzyme B, interferon γ (IFNγ), and interleukin 2 (IL-2) by CD8^+^ T cells was also effectively upregulated in the treated groups with DU102 and anti-PD-1 in comparison with the treated groups with DU102 or an-PD-1 alone (Fig. 7f–h). In summary, our results demonstrated that blocking Arf1 activity could enhance T-cell tumor infiltration through the LPE-PPARγ-NF-κB-CCL5-CCR5 pathway (Supplementary Fig. S7g), and the combined treatment of the Arf1 inhibitor and ICBs had synergistic ­anti-tumor effects.

## Discussion

The paucity of immune cells in tumors remains a major cause that the current immunotherapy is only effective in a small popu­lation of patients. Therefore, there is a pressing need to develop strategies to increase tumor antigen-specific CD8^+^ T-cell infiltration and turn “cold tumors” (such as colon and breast cancers) into “hot tumors” for effective immune therapy. In this study, we demonstrated that blocking the Arf1-mediated lipid metabolism in CSCs upregulated nuclear unsaturated fatty acids (LPE), which then sequestrated PPARγ from the PPARγ-NF-κB complex. The released NF-κB was then phosphorylated and translocated into the nucleus to regulate the transcription of chemokine CCL5. The CCL5-induced infiltration of cytotoxic T cells into tumors turned “cold” colon and breast cancers into “hot” ones. The new Arf1 inhibitors and PD-1 blockade synergistically and effectively reduced and eliminated tumors in melanoma grafts (Fig. 7).

It was previously demonstrated that lipid metabolism has an important role in regulating tumor immune response. Lipid changes in the TME are important for the interaction between cancer cells and immune cells [32]. Peroxidation of unsaturated fatty acids induces ferroptosis-mediated anticancer immune effects [33, 34], and IFNγ released from CD8^+^ T cells induces lipid peroxidation in tumor cells and promotes tumor regression [35, 36]. In our study, we found that inhibition of Arf1 in tumor cells significantly upregulated the unsaturated fatty acids (LPE). PPARγ is a nuclear transcription factor associated with the regulation of glucose metabolism and adipocyte differentiation. PPARγ binds a large number of compounds, including fatty acids or fatty acid derivatives derived from the diet or intracellular signaling pathways [37]. Here, we found that PPARγ formed a complex with either NF-κB or LPE in the cytoplasm. The increased level of LPE in the Arf1-deficient tumor cells sequestrated PPARγ from the PPARγ-NF-κB complex, which freed NF-κB for regulating CCL5 and T-cell tumor infiltration. Our previous TCGA data analysis also found an inverse correlation between Arf1 expression and T-cell infiltration and activation as well as better survival probability in various human cancers [15]. This information together suggests that inhibition of the Arf1-mediated lipid metabolic pathway in tumor cells has the effect of “one stone killing two birds”, that is, it not only kills CSCs but also alternates TME and activates systemic anti-tumor immunity. Targeting Arf1, LPE, or PPARγ may be a feasible new strategy to promote T-cell infiltration in tumors and improve tumor immunotherapy. The new Arf1 inhibitors described in this study can be used to convert previously immunological “cold tumors” into “hot ones” and may serve as a feasible new strategy of immunotherapy for such tumors either alone or in combination with current therapeutic approaches to achieve a better clinical outcome.

## Materials and methods

### Animals

Age-matched male and female mice were both utilized in all experiments. Six-week-old mice were used to start all investigations. All animals were maintained in specific pathogen-free ­facilities and all procedures were performed in accordance with the Animal Care and Use Committee at Fudan University.

### Cell culture

The colorectal carcinoma CT26 cells and the stage IV breast cancer 4T1 cells were cultured in RPMI1640 (SIGMA, Lot# RNBK0103) containing 10% fetal bovine serum (FBS) (HyClone, Cat# SH30396.03), 100 units/mL penicillin/streptomycin (Gibco, Cat# 15140122). The melanoma B16-F10 cells, the human embryonic kidney HEK 293T/17 cells, and the mouse hepatoma Hepa1-6 cells were cultured in DMEM (SIGMA, Lot# RNBK3466) supplemented with 10% FBS, 100 units/mL penicillin/streptomycin. All cells were grown in a humidified atmosphere containing 5% CO_2_ at 37°C. T cells were isolated from the spleen or lymph nodes (LNs) of 8–12-week-old mice after euthanasia by CO_2_. T cells were suspended and activated in a plate coated with 3 μg/mL anti-CD3 and anti-CD28 (InvivoMab) antibodies for 48 h. T cells were then cultured in RPMI1640 containing 10 ng/mL IL-2 (Genescript, Cat# Z02764) and 2-mercaptoethanol. Cell lines in this paper are shown in Supplementary Table S2.

### *In vivo* induced mouse models

For the MYC-ON liver cancer model, mice fed a doxycycline-­containing diet were switched to a normal diet at 6 weeks of age. After inducing liver tumor for 2 weeks, mice were treated with Arf1 inhibitors for 6 weeks and were euthanized with CO_2_ after 5 weeks.

### Engrafted tumor models

5 × 10^5^ CT26 cells or 5 × 10^5^ 4T1 cells were resuspended in sterile 1 × PBS and were subcutaneously injected into the BALB/c background mice. 5 × 10^5^ B16-F10 cells or 5 × 10^5^ Hepa1-6 cells were resuspended in sterile 1 × Phosphate-Buffered Saline (PBS) and were subcutaneously injected into the C57BL/6J background mice. Mice were treated with 5 mg/kg DU101 or DU102 or 1% Dimethyl sulfoxide (DMSO) by intragastric administration per day. Tumor volume was monitored every 2–3 days by a caliper and analyzed according to the formula: ½ ×longitudinal diameter (length) ×the greatest transverse diameter (width)^2^. Once tumor volume reached 2000 mm^3^, mice were euthanized by CO_2_ for 20 min. Mouse strains in this paper are shown in Supplementary Table S2.

### Generation of cell lines

Short hairpin RNA (shRNA) sequences are shown in Supplementary Table S1. The double shRNA oligonucleotides were annealed at 95°C for 20 min and were linked into the pLKO.1-Puro lentivirus vector. The targeting plasmid and packing plasmids, including pMD2.G and PsPax2, were co-transfected into HEK 293T/17 cells. The virus supernatant was harvested at 48 h and 72 h after transfection and filtered through a 0.22 μm filter. Targeting cells were transfected with lentivirus and fresh cell medium in a 1:1 ratio and were then selected by puromycin (Solarbio, Cat# IP1280) for 2 weeks. The lentiviral supernatant was stored at −80°C for a long time.

### Immunohistochemistry (IHC) and IF staining

Isolated tissues were fixed in 4% paraformaldehyde after mouse euthanasia by CO_2_ for 20 min. Gradient alcohols or 30% sucrose were used to dehydrate tissues. Dehydrated tissues were then embedded in paraffin wax or OCT (Tissue-Tek; Sakura Finetek USA, Cat# 4583), respectively. Five-micrometer paraffin sections were generated by a Leica RM2125 Microtome. Ten-micrometer frozen sections were generated by a Leica CM1950. For staining, in brief, paraffin sections were dewaxed with xylene and hydrated with gradient alcohol. Antigens were retrieved by sodium citrate solution (Solarbio, Cat# C1032) in a 100°C cooker for 25 min. Sections were washed in 1× PBS three times for 5 min each and blocked in 3% FBS for 1 h at room temperature. Sections were then incubated with primary antibodies at 4°C overnight. In the following day, sections were washed in 1× PBS three times for 5 min each. For IHC, paraffin sections were incubated with secondary antibodies for 1 h at room temperature and finally stained with DAB (3,3'-Diaminobenzidine Tetrahydrochloride) horseradish peroxidase color development kit (Beyotime, Cat# P0202). For IF staining, the frozen sections that had been incubated with primary antibodies were then stained with fluorescent secondary antibodies at room temperature and protected from light for 1 h. Sections were examined with the Olympus inverted fluorescence microscope (NEW IX73) or the Zeiss (LSM710) confocal.

### Fluorescence-activated cell sorting (FACS)

Cells were suspended in cell staining buffer (4abio, Cat# FXP005). For cell surface staining, conjugated fluorescent primary ­antibodies were diluted with cell staining buffer at 1:100, and 50 µL antibody dilution per sample was used for cell staining. They were incubated at room temperature in the dark for 1 h. For intracellular staining, cells were firstly fixed in 4% paraformaldehyde and permeabilized with 0.1% Triton X-100 (Beyotime, Cat# ST795). The intracellular staining was then performed according to corresponding protocols. DAPI (Sigma-Aldrich, Cat# D9542) or Zombie NIR Fixable kit (Biolegend, Cat# 423105) was used for identifying the live or dead cells. After staining, suspended cells were analyzed by flow cytometry. Flow cytometry was performed on LSR Fortessa (BD). Data were analyzed and presented with FlowJo_V10 software. Antibodies in this paper are shown in Supplementary Table S2.

### RNA extraction and quantitative real-time PCR (qRT-PCR)

Cells in the well were gently washed three times by cold 1× PBS, and total RNA was extracted by TRI Reagent® (Sigma-Aldrich, Cat# T9424). Complementary DNA (cDNA) was synthesized from 1 μg total RNA by the HiScript II Q Select RT SuperMix for qRT-PCR (Vazyme, Cat# R232-01). Primers for qRT-PCR are listed in Supplementary Table S1. The concentration of the extracted RNA was examined by NANO DROP ONE (Thermo Fisher Scientific). After cDNA was addressed with HiScript II One Step qRT-PCR SYBR Green Kit (Vazyme, Cat# Q221-01), the reaction was performed in a CFX96 Touch™ Real-Time PCR Detection System (Bio-Rad). The relative mRNA level was calculated by the 2^−ΔCT^ method and normalized by GAPDH.

### Plasmids

The pLKO.1-puro plasmid (Cat# 8453) was obtained from Addegene. The pET-21b (+) vector (Novagen, Cat# 69741) was used for protein expression. Mouse shRNA of Arf1, CCL5, PPARγ, and p65 was constructed in pLKO.1-puro vector after double digested by AgeI enzyme (New England Biolabs, Cat# R0552S) and EcoRI enzyme (New England Biolabs, Cat# R3101S), respectively. The gene fragments of p65 and PPARγ were built in pET-21b (+) vector digested by BamHI enzyme (New England Biolabs, Cat# R0136S) and XhoI enzyme (New England Biolabs, Cat# R0146S), respectively. T4 DNA ligase (New England Biolabs, Cat# R0101S) was applied for the linking reaction.

### Western blotting

Proteins were extracted from cells and mouse tissues with RIPA lysis buffer (Beyotime, Cat# P0013B) with PMSF (Beyotime, Cat# ST506). All protein expression levels were detected by western blotting. Briefly, equal amounts of proteins were size-fractionated on 10% SDS-PAGE. Proteins on the gels were transferred into nitrocellulose membranes and blocked with 5% non-fat milk for 1 h at room temperature. The membranes were first incubated with primary antibody at 4°C overnight on the shaker, then washed with 1× TBST (500 mmol/L NaCl, 20 mmol/L Tris-HCl (pH 7.5) and 0.1% Tween 20) three times, and incubated with the corresponding secondary antibody at room temperature for 1 h. After washing with TBST for three times, proteins were detected with BIO-RAD ChemiDoc Touch (America) after membranes were addressed with Omni-ECL™Femto Light Chemiluminescence Kit (Epizyme, Cat# SQ201L).

### ChIP-PCR assay

In the ChIP-PCR assay, 1% formaldehyde was used to cross-link the protein to DNA. Micrococcal nucleases were then added to digest cells to form small fragments of chromatin. After the immunoprecipitation (IP) reaction of antigen and antibody, DNA-protein crosslinking was removed after protease K and NaCl treatment, and DNA was recovered and detected by 1.8% agarose gel electrophoresis. The ChIP experiment was conducted with a ChIP assay kit (Sigma-Aldrich, Cat# 17-295) according to the manufacturer’s manual. Mouse anti-p65 antibody (CST, Cat# 6956) and mouse IgG antibody were used.

### IP

Cell lysed buffer contains 25 mmol/L Tris-HCl (pH 7.4), 150 mmol/L NaCl, 1% NP-40, 1 mmol/L EDTA, 5% glycerol, and protease inhibitor. Cells were washed with 1× PBS and lysed with the above buffer on ice. The cell lysates were transferred to a 1.5 mL centrifuge tube, and centrifuged at 13,000×*g* for 10 min. The supernatant was transferred to a new centrifuge tube. Protein concentration was detected by BCA Protein Assay Kit (Beyotime, Cat# P0012). An equal amount of protein was used in the IP experiment. Anti-p65 antibody (CST, Cat# 6956) or anti-PPARγ antibody (Proteintech, Cat# 16643-1-AP) was added into the lysates and incubated overnight at 4°C. Rabbit IgG antibody (1:100) was used as the control. Protein A+G Agarose (Beyotime, Cat# P2055) was applied to incubation with protein at 4°C for 1 h. The protein mixture was washed three times resuspended in protein loading buffer, and was finally examined by immunoblotting. Protein bands were visualized with Omni-ECL™Femto Light Chemiluminescence Kit (Epizyme, Cat# SQ201L) on BIO-RAD ChemiDoc Touch (America).

### T-cell isolation and culture

Spleen and LNs of wild-type mice were harvested by a 1 mL syringe plunger into a 40 μm cell strainer within RPMI 1640 containing 10% FBS, 100 units/mL penicillin/streptomycin. The cell suspension was centrifuged at 500 ×*g* at room temperature for 3 min and the RBC lysis buffer (Biolegend, Cat# 420301) was added into the pellet to break red blood cells at room temperature for 3 min. After centrifugation, T cells were resuspended in RPMI 1640 medium containing 10% FBS, 100 units/mL ­penicillin/streptomycin, 4 mmol/L L-glutamine (Gibco, Cat#25030081), 1 mmol/L sodium pyruvate (Gibco, Cat# 11360070), 50 μmol/L 2-mercaptoethanol (Sigma-Aldrich, Cat#M3180), 25 mmol/L HEPES (Gibco, Cat#15630130), 1 × MEM non-essential amino acids solution (Gibco, Cat#11140050). T-cell suspensions were placed in plates that had been incubated one day earlier with anti-CD3 and anti-CD28 antibodies at the final concentration of 3 μg/mL. Two days later, recombinant mouse IL-2 (Genescript, Cat# Z02764) with a final concentration of 10 ng/mL was added to the medium. Mouse T cells were cultured in a humidified atmosphere containing 5% CO_2_ at 37°C.

### T-cell chemotaxis assay

Tumor cells were treated with distinct reagents for 24 h and were then cultured in a normal medium for 24 h. Boyden transwell chambers were used to investigate T-cell trafficking by chemotaxis assay. The 5 μm transwell apparatus (JET BIOFIL, Cat# TCS004024) was placed on the tumor cells and T cells were placed in the upper chambers. After 50 min, T cells migrated into the lower chamber were harvested and stained with anti-CD3 antibody, anti-CCR5 antibody, and ZomBie Dye. The T-cell chemotaxis was analyzed by recording the cell counting per minute by flow cytometry.

### Enzyme-linked immunosorbent assay (ELISA)

Tumor cells were cultured in DMEM/RPMI1640 containing corresponding reagent for 24 h. Cells were washed with sterile 1× PBS and cultured in normal medium for one day. Finally, cell supernatants were collected and centrifuged at 4°C at 1000 rpm for 10 min. The level of chemokine CCL5 in cultured medium and mouse serum was detected by Mouse RANTES ELISA Kit (BOSTER, Cat# EK0495) according to the manufacturer’s instructions.

### Protein purification

To prepare the p65 protein for the corresponding assays, the gene fragment of p65 was ligated into the sites of the pET-21b (+) vector. After validating the correct sequence, the recombinant plasmids were transformed into BL21(DE3) competent cells (TRANS, Cat# CD601-02) for heterologous expression. When OD_600_ of pET-21b (+)/BL21 (DE3) in LB medium at 37°C reached 0.6, the isopropyl-β-D-thiogalactoside at a final concentration of 1 mmol/L was added and incubated at 16°C for 16 h. His-tagged proteins were extracted by protein purification kit (Beyotime, Cat# P2229S) and identified by western blotting.

### EMSA

The binding activity between CCL5 DNA and the purified PPARγ or NF-κB protein was assessed by EMSA according to the manufacturer’s introduction (Beyotime, Cat# GS009). The CCL5 sequences of the biotin-labeled oligonucleotide probe are listed in Supplementary Table S1. One microliter proteins were incubated with 2 μL of DNA, binding in the presence of a CCL5 biotin-labeled probe for 20 min at room temperature. The mixture was then separated by 4% non-denaturing PAGE and transferred onto a 0.45 μm nylon membrane (Beyotime, Cat# FFN13). Next, the membrane was exposed to a UV-light Crosslinker (CL-1000 UV Crosslinker, America) at 120 mJ/cm^2^ for 90 s. The membrane was incubated with a blocking buffer for 15 min before being incubated with a blocking buffer containing horseradish peroxidase-conjugated streptavidin (1:2000) for another 15 min. Having been washed with 1 × washing buffer for 5 min, the DNA-protein complex on the membrane was visualized by the ECL kit (Beyotime, Cat# GS009) on BIO-RAD ChemiDoc Touch (America).

### Nuclear lipidomic analysis

5 × 10^6^ Arf1-inhibited CT26 cells were washed with PBS three times and treated with 5 mL methyl tert-butyl ether at room temperature for 1 h on a shaker. The cells were then centrifuged at 4°C for 10 min at 1000×*g* after adding 1.25 mL H_2_O and mixed. The supernatant was transferred into a 1.5 mL tube and the samples were dried on the RapidVap (Labconco® RapidVap®). The dried samples were analyzed on the Sensitivity Triple Quadrupole/Composite Linear Ion Trap Liquid Chromatography Mass Spectrometry system (AB SCIEX QTRAP 5500+ LC-MS/MS).

### Quantification and statistical analysis

*In vitro*, triplicate samples and two independent assays were executed. *In vivo*, the number of mice used in the experiments was at least five mice. Student’s *t* test was applied to analyze the differences between the two groups. One-way ANOVA was used to compare more than two groups. Kaplan–Meier survival curves were generated and analyzed by two-tailed log-rank tests. Statistical analysis in cell assays and mouse experiments was performed by GraphPad Prism 9.0.0 software (GraphPad Software). *P* values of < 0.05 were considered statistically different. Error bars indicate mean ± SEM. The value of “*n*” represents the number of mice in the figure legends.

## Supplementary Material

load036_suppl_Supplementary_Figures_S1-S7_Tables_S1-S2

## Data Availability

All study data are included in the article and/or supplementary information. Materials are available upon request.
