# Supplementary material for: Blockade of Arf1-mediated lipid metabolism in cancers promotes tumor infiltration of cytotoxic T cells via the LPE-PPARγ-NF-κB-CCL5 pathway
Source: Life Metab. 2023 Sep 6;2(5):load036. doi: 10.1093/lifemeta/load036 (PMC11749100; doi:10.1093/lifemeta/load036)
Supplement: load036_suppl_Supplementary_Figures_S1-S7_Tables_S1-S2 [file load036_suppl_Supplementary_Figures_S1-S7_Tables_S1-S2.docx]

**Supplementary information**

**Blockade of Arf1-mediated lipid metabolism in cancers promotes tumor infiltration of cytotoxic T cells via the LPE-PPARγ-NF-κB-CCL5 pathway**

**Na Wang^1,‡^, Tiange Yao^1,‡^, Chenfei Luo^1^, Ling Sun^1^, Yuetong Wang^1,^*, Steven X. Hou^1,2,^***

^1^Department of Cell and Developmental Biology at School of Life Sciences, State Key Laboratory of Genetic Engineering, Institute of Metabolism and Integrative Biology, Human Phenome Institute, Fudan University, Shanghai 200438, China

**^‡^**These authors contributed equally to this work.

**^*^**Corresponding authors. Department of Cell and Developmental Biology at School of Life Sciences, State Key Laboratory of Genetic Engineering, Institute of Metabolism and Integrative Biology, Human Phenome Institute, Fudan University, Shanghai 200438, China. E-mail: [ytwang@fudan.edu.cn](mailto:ytwang@fudan.edu.cn) (Yuetong Wang ); stevenhou@fudan.edu.cn (Steven X. Hou )

^2^Lead contact

**
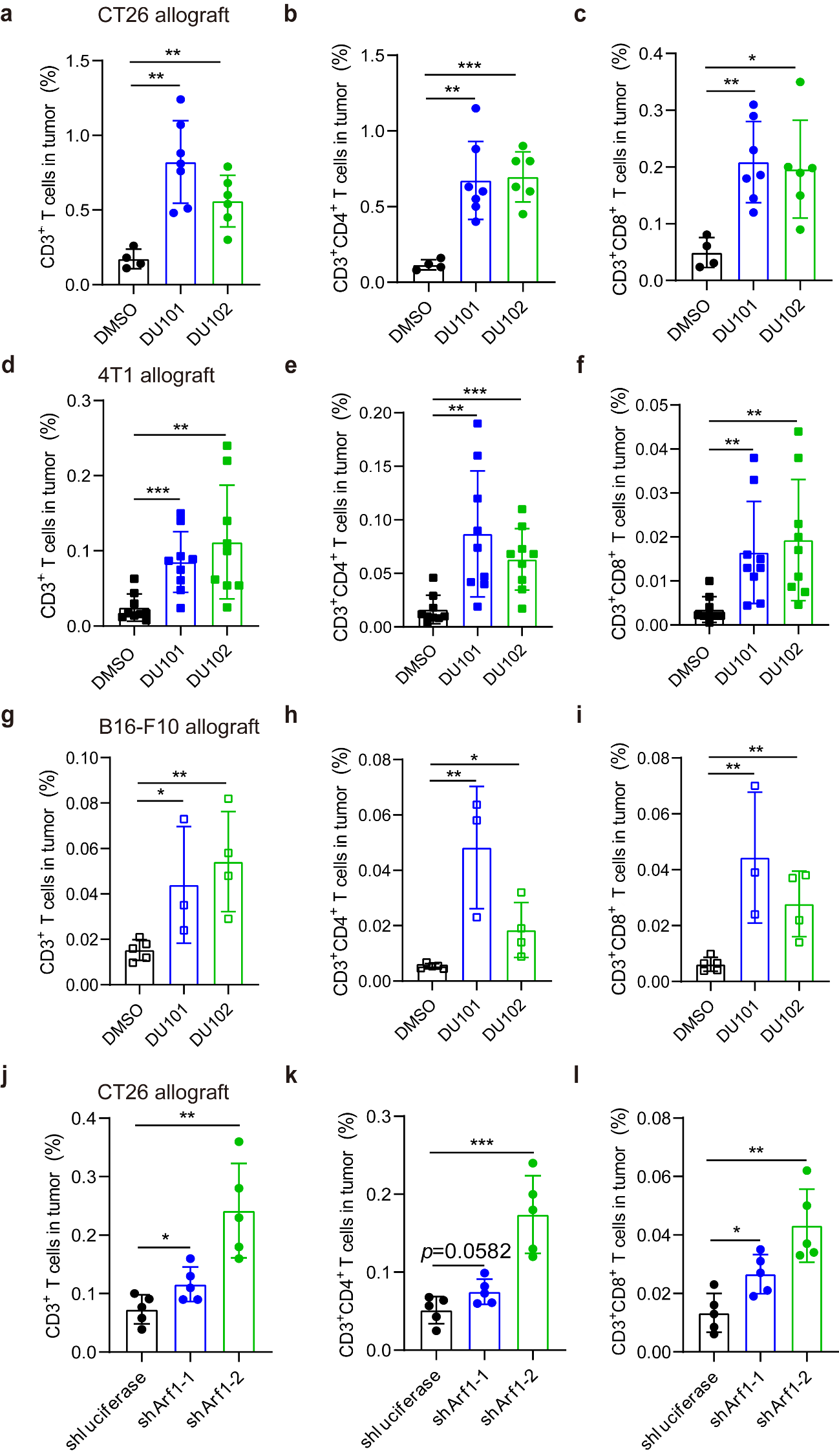
**

**Supplementary Figure S1** Blocking Arf1 induces T cell infiltration in multiple mouse tumors. (a−c) FACS analysis of CD3^+^ T cells (a), CD4^+^ T cells (b), and CD8^+^ T cells (c) in CT26 allografts treated with Arf1 inhibitors. (d−f) FACS analysis of CD3^+^ T cells (d), CD4^+^ T cells (e), and CD8^+^ T cells (f) in 4T1 allografts treated with Arf1 inhibitors. (g−i) FACS analysis of CD3^+^ T cells (g), CD4^+^ T cells (h), and CD8^+^ T cells (i) in B16-F10 melanoma treated with Arf1 inhibitors. (j−l) FACS analysis of CD3^+^ T cells (j), CD4^+^ T cells (k), and CD8^+^ T cells (l) in Arf1-deficient CT26 allografts. Data are shown as mean ± SEM. Student’s *t* test. ^*^*P*< 0.05, ^**^*P*< 0.01, ^***^*P*< 0.001.


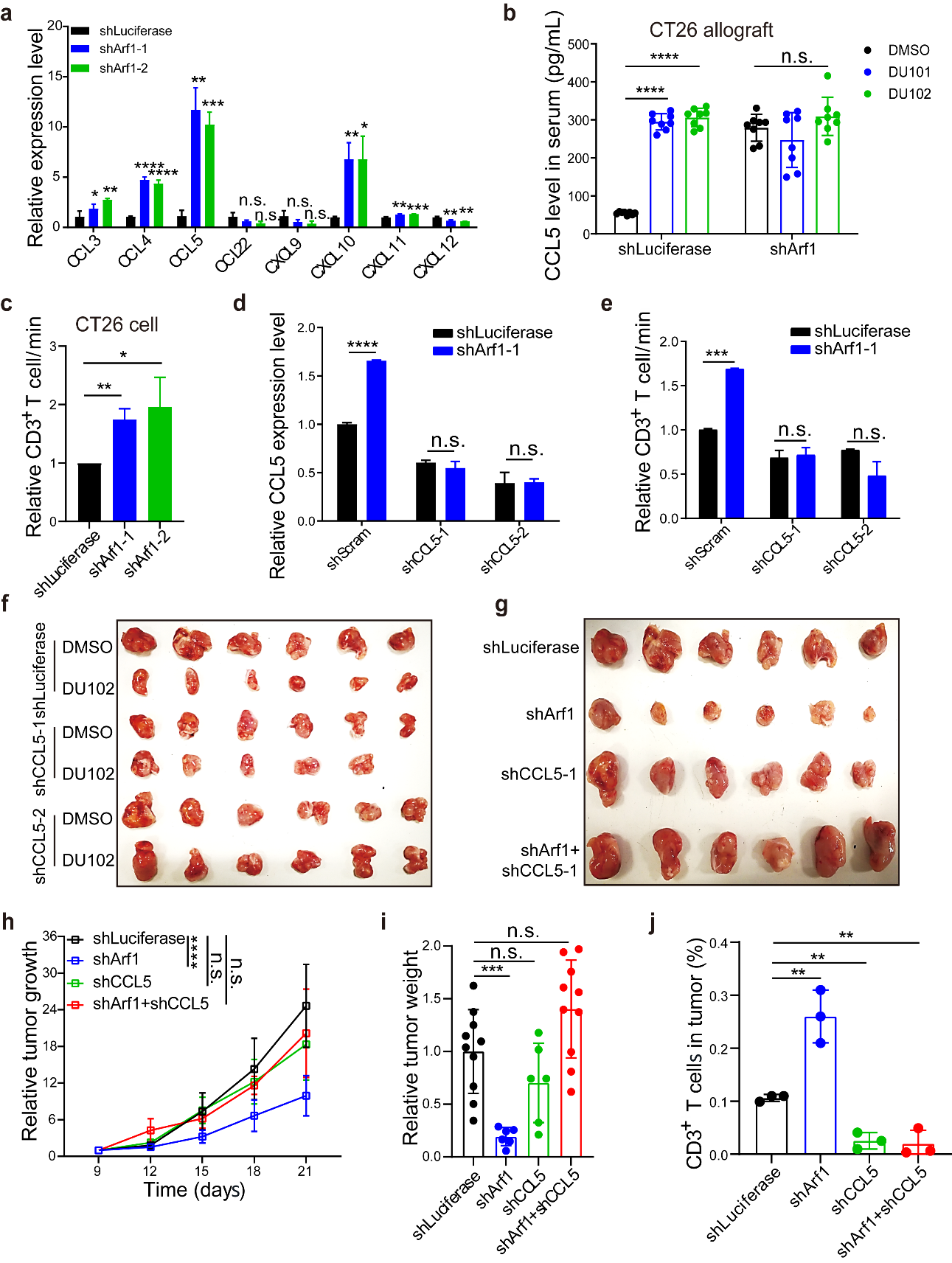


**Supplementary Figure S2** Chemokine CCL5 mediates T cell recruitment in Arf1-inhibited tumors. (a) The mRNA levels of the indicated chemokines in Arf1-deficient CT26 cells were measured by qRT-PCR. (b) Serum CCL5 levels in CT26 allografts with the indicated knockdowns and treatments were measured by ELISA assay (*n* = 8 in each group). (c) FACS analysis of relative percentage of CD3^+^ T cells in Arf1-deficient CT26 cells. (d) The relative mRNA levels of CCL5 in CT26 cells with the indicated knockdowns were measured by qRT-PCR. (e) The relative percentages of infiltrated CD3^+^ T cells in CT26 cells with the indicated knockdowns were analyzed by FACS. (f) The tumor images of CT26 allografts with the indicated knockdowns and treatments (*n* = 6 in each group). (g) The tumor pictures of CT26 allografts with the indicated knockdowns (*n* = 6 in each group). (h and i) The relative tumor volumes (h) and tumor weights (i) of CT26 allografts with the indicated knockdowns (*n* = 6 in each group). (j) The percentages of CD3^+^ T cells in CT26 allografts with the indicated knockdowns (*n* = 3 in each group). Data are shown as mean ± SEM. Student’s *t* test. ^*^*P* < 0.05, ^**^*P* < 0.01, ^***^*P* < 0.001, ^****^*P* < 0.0001. n.s., no significance.


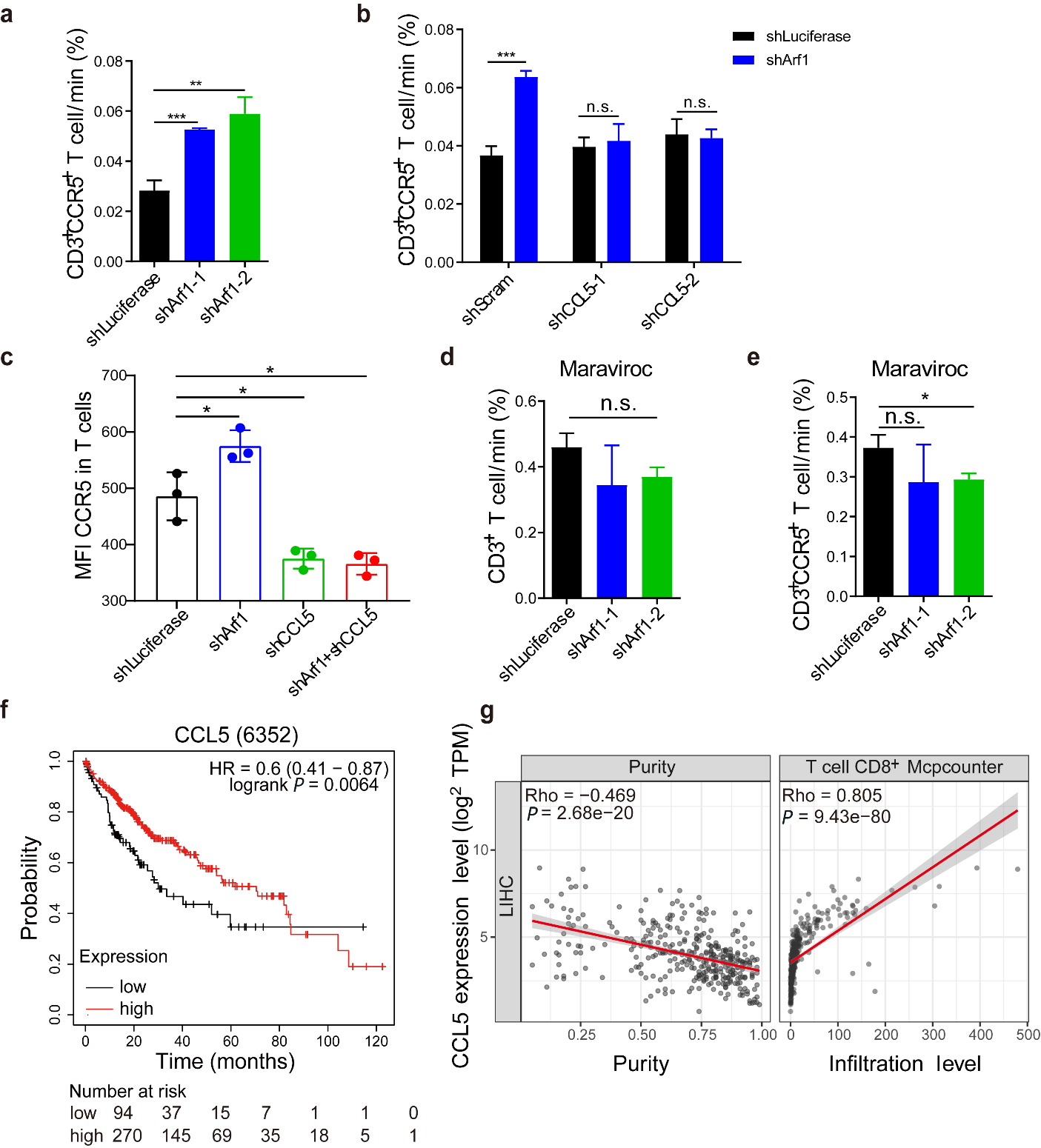


**Supplementary Figure S3** Arf1 inhibition promotes T cell infiltration through the CCL5-CCR5 pathway. (a) The proportions of CD3^+^CCR5^+^ T cells in CT26 cells with the indicated knockdowns were examined by FACS. (b) FACS analysis of the percentage of CD3^+^CCR5^+^ T cells in CT26 cells with the Arf1 indicated knockdowns. (c) CCR5 mean fluorescent intensities (MFI) in infiltrating T cells of CT26 allografts with the indicated knockdowns (*n* = 3 in each group). (d and e) T cells were pretreated with Maraviroc for one day, and the infiltrated CD3^+^ T cells (d) and CD3^+^CCR5^+^ T cells (e) were analyzed by FACS. (f) The correlation analysis between the survival curve and CCL5 levels in patients with liver hepatocellular carcinoma (LIHC) in TCGA database. (g) The correlation analysis between CCL5 and CD8^+^ T cell infiltration in patients with LIHC from TCGA database. Data are shown as mean ± SEM. Student’s *t* test. ^*^*P*< 0.05, ^**^*P*< 0.01, ^***^*P*< 0.001. n.s., no significance.


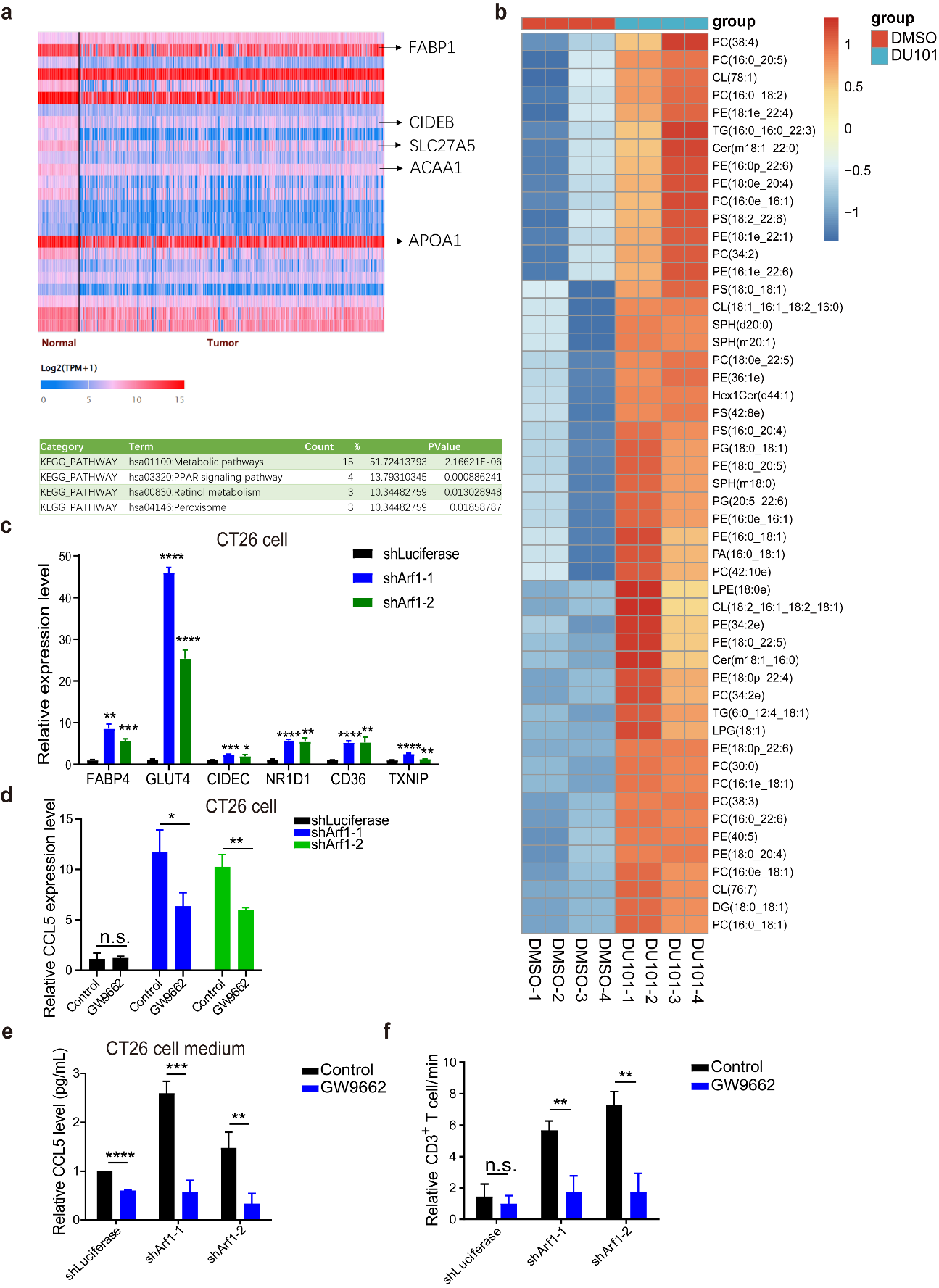


**Supplementary Figure S4** Arf1 inhibition promotes CCL5 transcription through the LPE-PPARγ axis. (a) Correlation analysis between Arf1 and PPARγ pathway in TCGA database. (b) Nuclear lipidomics analysis in CT26 cells with DMSO or the Arf1 inhibitor treatment. (c) The mRNA expression of downstream genes of PPARγ in the Arf1-deficient CT26 cells was measured by qRT-PCR. (d) The CCL5 mRNA levels in the Arf1-deficient CT26 cells with GW9662 treatment were quantified by qPCR. (e) The CCL5 levels in cell medium collected from the Arf1-deficient CT26 cells treated with GW9662 were determined by CCL5 ELISA assay. (f) FACS analysis of CD3^+^ T cell migration in the Arf1-deficient CT26 cells treated with GW9662. Data are shown as mean ± SEM. Student’s *t* test. ^*^*P* < 0.05, ^**^*P* < 0.01, ^***^*P* < 0.001, ^****^*P* < 0.0001. n.s., no significance.

**
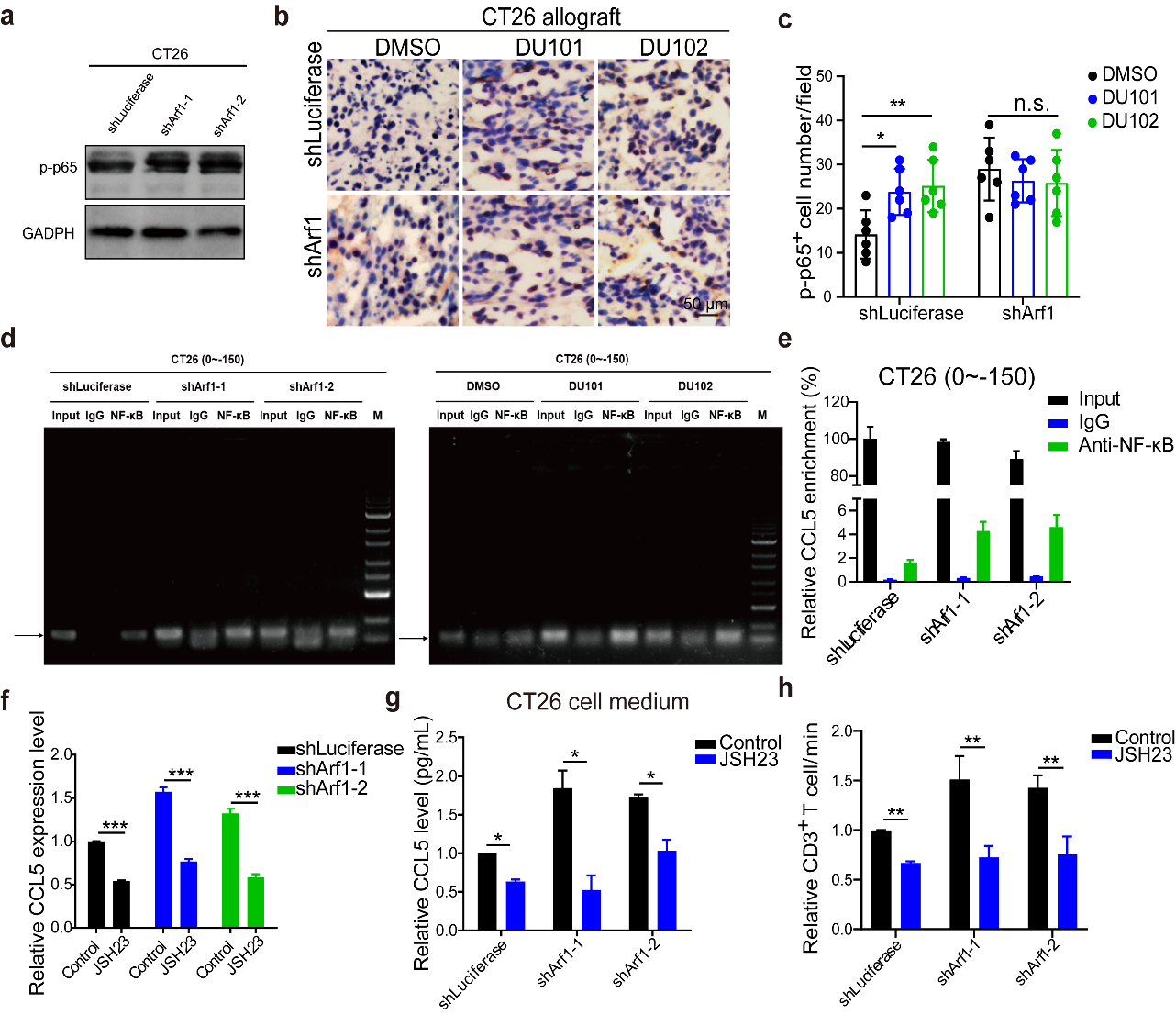
**

**Supplementary Figure S5** Arf1 inhibition activates the NF-κB pathway. (a) The phosphorylated p65 was increased in the Arf1-deficient CT26 cells. (b) The phosphorylated p65 was examined in CT26 allografts with the indicated knockdowns and treatments by IHC staining. (c) The quantification of p-p65^+^ cells per field. (d) The pictures of ChIP-PCR agarose gel electrophoresis using cell lysates with the indicated treatments. (e) The relative CCL5 enrichments were detected by ChIP-qPCR using cell lysates with the indicated knockdowns and treatments. (f) The CCL5 mRNA levels were evaluated in CT26 cells with the indicated knockdowns and treatments by qRT-PCR. (g) The CCL5 levels in cell medium collected from CT26 cells with the indicated knockdowns and treatments were detected by CCL5 ELISA assay. (h) FACS analysis of the relative percentages of CD3^+^ T cell infiltration in CT26 cells with the indicated knockdowns and treatments. Data are shown as mean ± SEM. Student’s *t* test. ^*^*P* < 0.05, ^**^*P* < 0.01, ^***^*P* < 0.001. n.s., no significance.


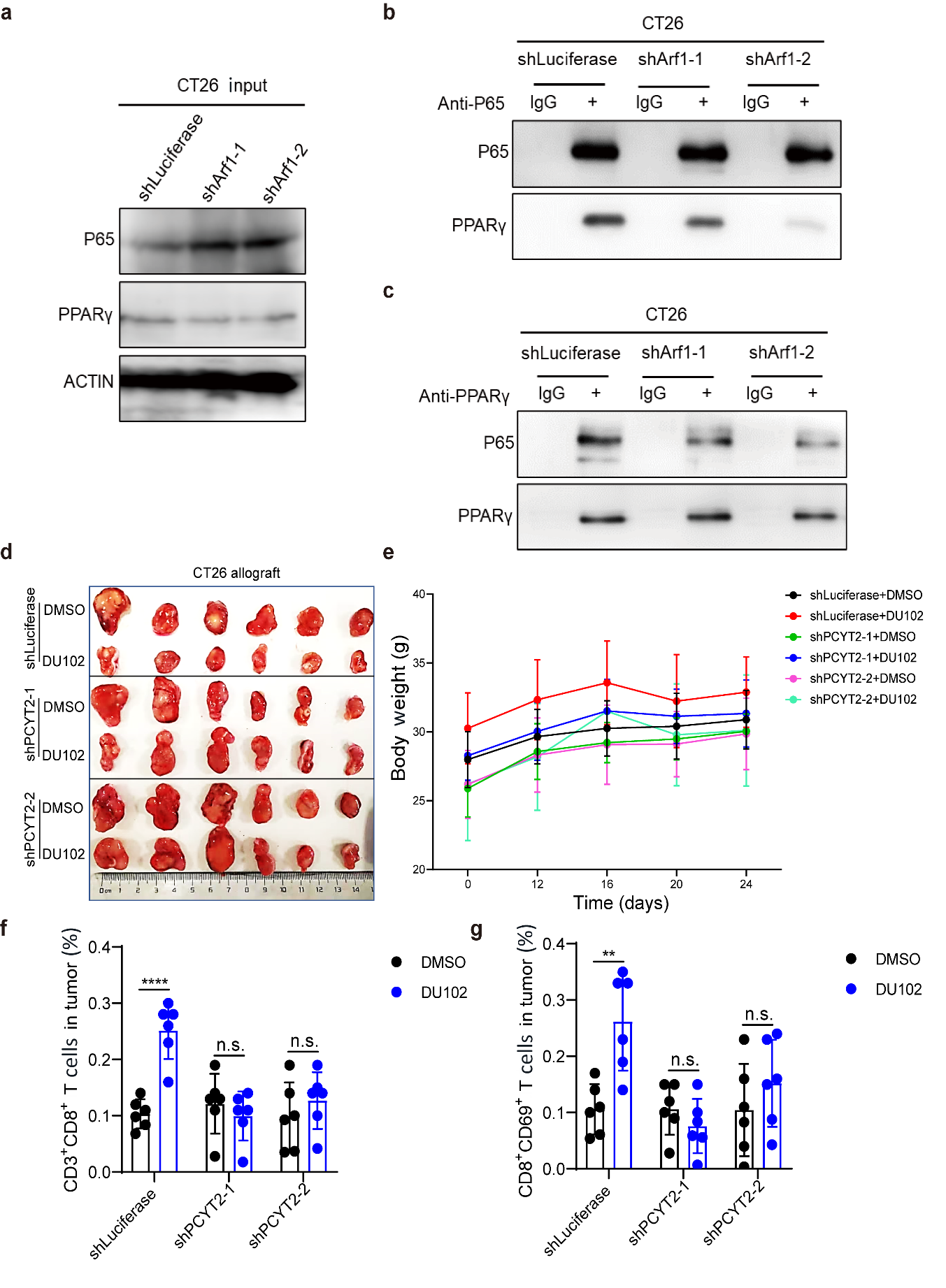


**Supplementary Figure S6** Arf1 blockade disrupts the interaction between PPARγ and NF-κB. (a−c) The interaction between p65 and PPARγ in the Arf1-deficient CT26 cells was examined by co-IP experiments. The protein levels in input (a), IP with anti-P65 antibody (b), and IP with anti-PPARγ antibody (c) were detected by immunoblotting. (d) The images of CT26 allografts with the indicated knockdowns and treatments (*n* = 6 each group). (e) Body weights of the PCYT2-deficient CT26 allografts with DMSO or DU102 treatment (*n* = 6 in each group). (f) FACS analysis of CD3^+^CD8^+^ T cells in CT26 allografts with the indicated knockdowns and treatments (*n* = 6 in each group). (g) FACS analysis of CD8^+^CD69^+^ T cells in CT26 allografts with the indicated knockdowns and treatments (*n* = 6 in each group). Data are shown as mean ± SEM. Student’s *t* test. ^**^*P* < 0.01, ^***^*P* < 0.001. n.s., no significance.


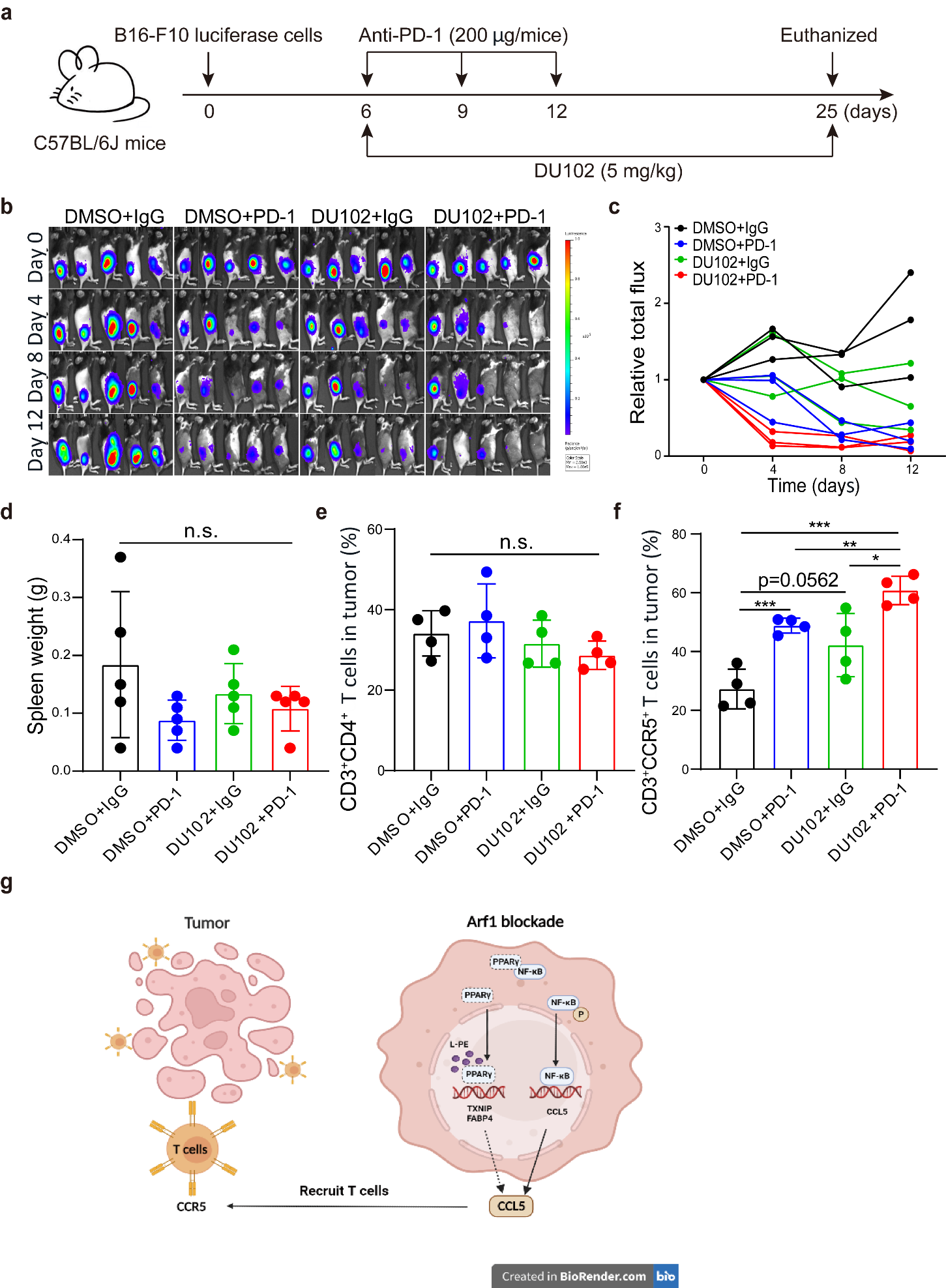


**Supplementary Figure S7** The Arf1 inhibitor enhances the anti-tumor activity of anti-PD-1 antibody. (a) Experimental design for combination treatment of Arf1 inhibitor and anti-PD-1 antibody. (b and c) Bioluminescence images and quantification of relative total fluorescence of mice with B16-F10 allograft with the indicated treatments (*n* = 5 in each group). (d) Spleen weights of mice subcutaneously inoculated with B16-F10 cells and the indicated treatments (*n* = 5 in each group). (e and f) FACS analysis of CD3^+^CD4^+^ T cells and CD3^+^CCR5^+^ T cells in tumors. (g) Model of the Arf1 blockade induced the LPE- PPARγ-NF-κB-CCL5 pathway. Data are shown as mean ± SEM. Student’s *t* test. ^*^*P* < 0.05, ^****^*P* < 0.0001. n.s., no significance.

**Supplementary Table S1 The list of primers in this study.**

| **Primer** | **Sequence** |
| --- | --- |
| Q-mCCL3-F | 5’-AGTTCTCTGCATCACTTGCTG-3’ |
| Q-mCCL3-R | 5’-CGGCTTCGCTTGGTTAGGAA-3’ |
| Q-mCCL4-F | 5’-CTGTGCTGATCCCAGTGAATC-3’ |
| Q-mCCL4-R | 5’-TCAGTTCAGTTCCAGGTCATACA-3’ |
| Q-mCCL5-F | 5’-GCTGCTTTGCCTACCTCTCC-3’ |
| Q-mCCL5-R | 5’-TCGAGTGACAAACACGACTGC-3’ |
| Q-mCCL22-F | 5’-ATTACGTCCGTTACCGTCTGC-3’ |
| Q-mCCL22-R | 5’-TCCCTGAAGGTTAGCAACACC-3’ |
| Q-mCXCL9-F | 5’-CCAGTAGTGAGAAAGGGTCGC-3’ |
| Q-mCXCL9-R | 5’-AGGGCTTGGGGCAAATTGTT-3’ |
| Q-mCXCL10-F | 5’-GTGGCATTCAAGGAGTACCTC-3’ |
| Q-mCXCL10-R | 5’-TGATGGCCTTCGATTCTGGATT-3’ |
| Q-mCXCL11-F | 5’-GACGCTGTCTTTGCATAGGC-3’ |
| Q-mCXCL11-R | 5’-GGATTTAGGCATCGTTGTCCTTT-3’ |
| Q-mCXCL12-F | 5’-ATTCTCAACACTCCAAACTGTGC-3’ |
| Q-mCXCL12-R | 5’-ACTTTAGCTTCGGGTCAATGC-3’ |
| Q-mTXNIP-F | 5’-GTTGCGTAGACTACTGGGTGAAG-3’ |
| Q-mTXNIP-R | 5’-CTCCTTTTTGGCAGACACTGGTG-3’ |
| Q-mNR1D1-F | 5’-CAGGCTTCCGTGACCTTTCTCA-3’ |
| Q-mNR1D1-R | 5’-TAGGTTGTGCGGCTCAGGAACA-3’ |
| Q-mFABP4-F | 5’-TGAAATCACCGCAGACGACAGG-3’ |
| Q-mFABP4-R | 5’-GCTTGTCACCATCTCGTTTTCTC-3’ |
| Q-mGLUT4-F | 5’-GGTGTGGTCAATACGGTCTTCAC-3’ |
| Q-mGLUT4-R | 5’-AGCAGAGCCACGGTCATCAAGA-3’ |
| Q-mCIDEC-F | 5’-TCGGAAGGTTCGCAAAGGCATC-3’ |
| Q-mCIDEC-R | 5’-CTCCACGATTGTGCCATCTTCC-3’ |
| Q-mCD36-F | 5’-AAGTTGTCCTTGAAGAAG-3’ |
| Q-mCD36-R | 5’-AGATAACGAACTCTGTATGTG-3’ |
| Q-mArf1-F | 5’-GGGGTCGTTGATGGCAACA -3’ |
| Q-mArf1-R | 5’-AGGTCGGTGTGAACGGATTTG -3’ |
| Q-m18S-F | 5’-AGGGGAGAGCGGGTAAGAGA -3’ |
| Q-m18S-R | 5’-GGACAGGACTAGGCGGAACA -3’ |
| Q-mGAPDH-F | 5’-GGGGTCGTTGATGGCAACA -3’ |
| Q-mGAPDH-R | 5’-AGGTCGGTGTGAACGGATTTG-3’ |
| Q-PPARγ-F | 5’-TTGCTGTGGGGATGTCTCAC-3’ |
| Q-mPPARγ-R | 5’-AACAGCTTCTCCTTCTCGGC-3’ |
| Q-mPCYT2-F | 5’-CGATGGCTGCTATGACATGGT-3’ |
| Q-mPCYT2-R | 5’-GCCCCTTATGCTTGGCAATCT-3’ |
| CCL5-probe-F | 5’-GTCTTTTGTGGAAACTCCCCAAG-3’ |
| CCL5-probe-R | 5’-CTTGGGGAGTTTCCACAAAAGAC-3’ |
| M-shArf1-F-1 | 5’-CCGGGGAATATCTTTGCAAACCTCT CTCGAGAGAGGTTTGCAAAGATATTCCTTTTTG-3’ |
| M-shArf1-R-1 | 5’-AATTCAAAAAGGAATATCTTTGCAAACCTCT CTCGAG AGAGGTTTGCAAAGATATTCC-3’ |
| M-shArf1-F-2 | 5’-CCGGGCGAAATTGTGACCACCATTCCTCGAG GAATGGTGGTCACAATTTCGC TTTTTG-3’ |
| M-shArf1-R-2 | 5’-AATTCAAAAAGCGAAATTGTGACCACCATTC CTCGAG GAATGGTGGTCACAATTTCGC-3’ |
| M-shCCL5-F-1 | 5’-CCGGGCCCACGTCAAGGAGTATTTCCTCGAG GAAATACTCCTTGACGTGGGC TTTTTG-3’ |
| M-shCCL5-R-1 | 5’-AATTCAAAAAGCCCACGTCAAGGAGTATTTC CTCGAG GAAATACTCCTTGACGTGGGC-3’ |
| M-shCCL5-F-2 | 5’-CCGGCCAGAGAAGAAGTGGGTTCAACTCGAG TTGAACCCACTTCTTCTCTGG TTTTTG-3’ |
| M-shCCL5-R-2 | 5’-AATTCAAAAACCAGAGAAGAAGTGGGTTCAA CTCGAG TTGAACCCACTTCTTCTCTGG-3’ |
| M-shPCYT2-F-1 | 5’-CCGG CCGGGAATATGCTGACAGTTT CTCGAG AAACTGTCAGCATATTCCCGG TTTTTG-3’ |
| M-shPCYT2-R-1 | 5’-AATTCAAAAA CCGGGAATATGCTGACAGTTT CTCGAG AAACTGTCAGCATATTCCCGG-3’ |
| M-shPCYT2-F-2 | 5’-CCGG CTGGAGTATGAAGCACGGAAT CTCGAG ATTCCGTGCTTCATACTCCAG TTTTTG-3’ |
| M-shPCYT2-R-2 | 5’-AATTCAAAAA CTGGAGTATGAAGCACGGAAT CTCGAG ATTCCGTGCTTCATACTCCAG-3’ |

**Supplementary Table S2 The list of regents and antibodies used in this study.**

| **Antibodies Source Identifier** | | |
| --- | --- | --- |
| Rat anti-mouse CD3-APC | Biolegend | Cat# 100236 |
| Rat anti-mouse CD4-FITC | Biolegend | Cat# 100405 |
| Rat anti-mouse CD8-Pacific Blue | Biolegend | Cat# 100725 |
| Armenian Hamster anti-mouse CCR5- PerCP/Cyanine5.5 | Biolegend | Cat# 107015 |
| Zombie NIR™ Fixable Viability Kit | Biolegend | Cat# 423106 |
| Donkey anti-mouse IgG H&L -Alexa Fluor® 568 | Abcam | Cat# ab175700 |
| Goat anti-mouse IgG H&L-HRP | Abcam | Cat# ab6789 |
| Goat anti-rabbit IgG H&L-HRP | Abcam | Cat# ab6721 |
| Rabbit anti-Arf1 antibody | Thermo Fisher Scientific | Cat# PA1-127 |
| Rabbit anti-H3 antibody | Cell Signaling Technology | Cat# 9715S |
| Rabbit anti-β-Actin antibody | Cell Signaling Technology | Cat# 4967S |
| Mouse anti-GAPDH antibody | Thermo Fisher Scientific | Cat# MA5-15738 |
| Mouse anti-IgG antibody | Cell Signaling Technology | Cat# 5415S |
| Mouse anti-NF-κB p65 (L8F6) antibody | Cell Signaling Technology | Cat# 6956T |
| Rabbit anti-PPARγ antibody | Proteintech | Cat# 16643-1-AP |
| Rabbit anti-Phospho- NF-κB p65 (Ser536) antibody | Thermo Fisher Scientific | Cat# MA5-15160 |
| anti-mouse CD3ε antibody | InVivoMAb | Cat# BE0001-1 |
| anti-mouse CD28 antibody | InVivoMAb | Cat# BE0015-1 |
| InVivoMAb polyclonal Armenian hamster IgG | InVivoMAb | Cat# BE0091 |
| InVivoMAb anti-mouse PD-1 (CD279) | InVivoMAb | Cat# BE0033-2 |
| **Buffers, chemicals, peptides, and recombinant proteins** | | |
| Brefeldin A | Selleck | Cat# S7046 |
| Golgicide A | Selleck | Cat# S7266 |
| JSH23 | Selleck | Cat# S7351 |
| GW9662 | Selleck | Cat# S2915 |
| Maraviroc | Selleck | Cat# S2003 |
| DAPI | Sigma-Aldrich | Cat# D9542 |
| ReadyShield® protease and phosphatase inhibitor cocktail | Sigma-Aldrich | Cat# PPC2020 |
| Puromycin Dihydrochloride | Solarbio | Cat# IP1280 |
| Recombinant Mouse PPARγ protein | COSMO BIO | Cat# CSB-EP018424MOb0 |
| Recombinant Human IL-2 protein | Genescript | Cat# Z00368 |
| **Critical commercial assays** | | |
| Protein A+G Agarose | Beyotime | Cat# P2055 |
| RBC Lysis Buffer (10**╳**) | Biolegend | Cat# 420301 |
| Mouse Rantes ELISA Kit PicoKine® | BOSTER | Cat# EK0495 |
| Chemiluminescent EMSA Kit | Beyotime | Cat# GS009 |
| Omni-ECL™Femto Light Chemiluminescence Kit | Epizyme | Cat# SQ201L |
| His-tag Protein Purification Kit | Beyotime | Cat# P2229S |
| Agarose ChIP Kit | Sigma-Aldrich | Cat# 17-295 |
| BCA Protein Assay Kit | Beyotime | Cat# P0012 |
| **Cell lines** | | |
| CT26 | ATCC | Cat# CRL-2638 |
| 4T1 | ATCC | Cat# CRL-2539 |
| B16-F10 | ATCC | Cat# CRL-6475 |
| Hepa1-6 | ATCC | Cat# CRL-1830 |
| HEK 239T/17 | ATCC | Cat# CRL-11268 |
| **Organisms/strains** | | |
| Mouse: C57BL/6J | GemPharmatech | Strain NO. N00013 |
| Mouse: BALB/c | GemPharmatech | Strain NO. N00020 |
| Mouse: B6.Cg-Tg(Cebpb-tTA)5Bjd/J | The Jackson  Laboratory | Strain #: 003563  IMSR_JAX:003563 |
| Mouse: FVB/N-Tg(tetO-MYC)36aBop/J | The Jackson  Laboratory | Strain #: 019376  IMSR_JAX:019376 |
| **Recombinant DNA** | | |
| pET-21+(b)-P65-His | This paper | N/A |
| **Bacterial and virus strains** |  |  |
| BL21(DE3) | Vazyme | Cat# C504-02 |
| DH5α | Vazyme | Cat# C502-02/03 |
| **Software and algorithms** | | |
| FlowJo_V10 | Becton Dickinson | https://www.flowjo.com/solutions/flowjo |
| GraphPad Prism 9.0.0 | GraphPad | https://www.graphpad.com/ |

**Database analysis**

The correlation between CCL5 expression level and survive time of LIHC patients was analyzed at the website of [Kaplan-Meier plotter [Liver RNAseq] (kmplot.com)](http://kmplot.com/analysis/index.php?p=service&cancer=liver_rnaseq) (38). The correlation of CCL5 expression level with CD8^+^ T cell infiltration was analyzed at the website of [TIMER2.0 (comp-genomics.org)](http://timer.comp-genomics.org/) (39). Top co-dependencies of Arf1 were analyzed at the website of [ualcan.path.uab.edu/home](http://ualcan.path.uab.edu/index.html) (40), and these negative co-dependencies were further used for KEGG analysis at the website of [KEGG: Kyoto Encyclopedia of Genes and Genomes](https://www.genome.jp/kegg/).

**Supplemental References**

[38] O. Menyhart, A. Nagy, B. Gyorffy. Determining consistent prognostic biomarkers of overall survival and vascular invasion in hepatocellular carcinoma. *R Soc Open Sci* 2018; 5:181006.

[39] T. Li et al. TIMER2.0 for analysis of tumor-infiltrating immune cells. *Nucleic Acids Res* 2020; 48:W509-W514.

[40] D.S. Chandrashekar et al. UALCAN: An update to the integrated cancer data analysis platform. *Neoplasia* 2022; 25:18-27.
